# The Bidirectional Interplay of α-Synuclein with Lipids in the Central Nervous System and Its Implications for the Pathogenesis of Parkinson’s Disease

**DOI:** 10.3390/ijms241713270

**Published:** 2023-08-26

**Authors:** Kristina Battis, Wei Xiang, Jürgen Winkler

**Affiliations:** Department of Molecular Neurology, University Hospital Erlangen, Friedrich-Alexander-University Erlangen-Nürnberg, 91054 Erlangen, Germany; kristina.battis@uk-erlangen.de (K.B.); wei.xiang@uk-erlangen.de (W.X.)

**Keywords:** α-synuclein, lipids, Parkinson’s disease, post-translational modification

## Abstract

The alteration and aggregation of alpha-synuclein (α-syn) play a crucial role in neurodegenerative diseases collectively termed as synucleinopathies, including Parkinson’s disease (PD). The bidirectional interaction of α-syn with lipids and biomembranes impacts not only α-syn aggregation but also lipid homeostasis. Indeed, lipid composition and metabolism are severely perturbed in PD. One explanation for lipid-associated alterations may involve structural changes in α-syn, caused, for example, by missense mutations in the lipid-binding region of α-syn as well as post-translational modifications such as phosphorylation, acetylation, nitration, ubiquitination, truncation, glycosylation, and glycation. Notably, different strategies targeting the α-syn-lipid interaction have been identified and are able to reduce α-syn pathology. These approaches include the modulation of post-translational modifications aiming to reduce the aggregation of α-syn and modify its binding properties to lipid membranes. Furthermore, targeting enzymes involved in various steps of lipid metabolism and exploring the neuroprotective potential of lipids themselves have emerged as novel therapeutic approaches. Taken together, this review focuses on the bidirectional crosstalk of α-syn and lipids and how alterations of this interaction affect PD and thereby open a window for therapeutic interventions.

## 1. Introduction

Alpha-synuclein (α-syn) is a small 14-kDa protein first discovered by Maroteaux and colleagues in 1988 [1]. In the central nervous system (CNS), α-syn is expressed abundantly in neurons [2], while it is also present in the peripheral nervous system (PNS), gut, muscle, liver, heart, lungs, kidney, and blood cells [3,4]. Since α-syn is enriched in presynaptic terminals and associated with synaptic vesicles, a large number of studies indicates the important role of α-syn in neurotransmission and synaptic plasticity [5,6,7]. In addition, α-syn plays a role in transcriptional regulation of dopamine synthesis [8]. Diverse physiological forms of α-syn have been reported: the protein may exist as unstructured monomer [9,10], α-helical monomer or multimer [11,12] that interacts with biological membranes [13]. Pathological alterations in the α-syn structure are predominantly linked to its abnormal aggregation from monomers to oligomeric or fibrillar species [11,14] showing varying degrees of assembly, solubility, and pathogenicity [15]. Neurotoxic effects of aggregated α-syn are multifaceted. For example, addition of α-syn oligomers to primary neurons in culture induces reactive oxygen species, increases cytosolic calcium, disrupts membranes, and thus leads to cell death [15,16]. Furthermore, exposure of neurons to fibrillar forms of α-syn induces prion-like propagation of α-syn aggregation, resulting in the formation of inclusions that are morphologically and biochemically similar to those detected in diseased brains [17,18]. Diseases associated with α-syn aggregation are collectively termed synucleinopathies, consisting of Parkinson’s disease (PD), multiple system atrophy (MSA), and Lewy body disease [19].

**Figure 1:**
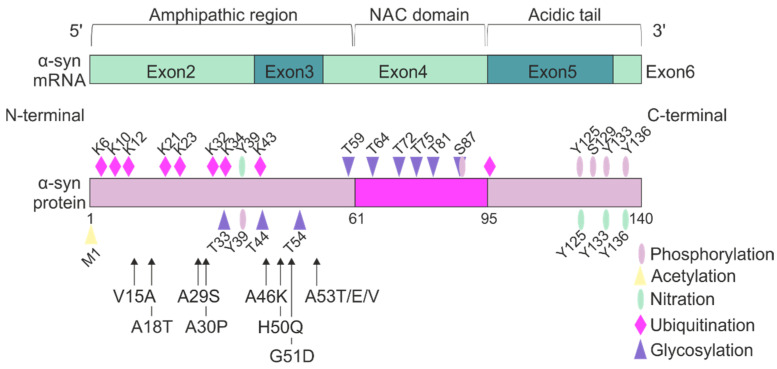
Structure of alpha-synuclein (α-syn). α-syn is encoded by the *SNCA* gene. This gene is transcribed into mRNA consisting of five exons. Following translation, the protein consists of distinct domains: the amphipathic region at the N-terminus, the non-amyloid-β component (NAC) domain, and the C-terminal acidic tail. Moreover, α-syn undergoes a variety of post-translational modifications (PTMs), including phosphorylation, acetylation, nitration, ubiquitination, truncation, glycosylation, and glycation. Monogenic PD-associated point mutations are indicated with arrows and are exclusively present in the N-terminal amphipathic region.

PD is the most prevalent neurodegenerative movement disorder and is clinically characterized by motor deficits such as bradykinesia, rigidity, and resting tremor [20,21]. The neuropathological hallmarks of PD are the formation of Lewy bodies or Lewy neurites containing aggregated α-syn accompanied with a substantial loss of dopaminergic neurons in the substantia nigra [22,23]. The involvement of α-syn in dopaminergic neuronal cell death was suggested after the identification of the first missense mutations (A30P, E46K, and A53T) in the α-syn encoding gene, *SNCA*, linked to monogenic PD phenotypes [22,24,25]. The primary structure of α-syn is composed of three well-described domains that determine its biological functions: the N-terminal amphipathic region [26,27], responsible for lipid binding [27]; the central hydrophobic non-amyloid-β component (NAC) region [28], important for aggregation of the protein [29,30] as well as axonal transport [31]; and the acidic C-terminal domain [32], decisive for protein interactions [33] and oligomerization of the protein [34] (Figure 1). Interestingly, all missense mutations linked so far to familial forms of PD (e.g., A30P, E46K, H50Q, G51D, A53E, and A53T) reside in the lipid-interacting N-terminal domain of α-syn [22,24,25,35] (Figure 1). Thus, these mutations may represent a functional “hot spot” resulting in a detrimental impact on the lipid-binding properties of α-syn and its physiological function. Moreover, α-syn conformation and aggregation propensity may be consequently changed after exposure to distinct lipid classes [36].

Lipids play an essential role in the CNS. Besides ensuring compartmentalization of cells and organelles through the formation of lipid-rich membranes, lipids act as bioactive signaling molecules [37]. Furthermore, they participate in mitochondrial metabolism [37]. In the CNS, lipids are predominantly metabolized in neurons and astrocytes [37]. However, lipids are one of the main components of myelin sheaths generated by oligodendrocytes and thus present a major player in lipid metabolism as well [38]. Furthermore, there is a physiological interaction of α-syn with different lipid classes, especially biomembranes, [27,39,40] and lipids are dysregulated in PD [41]. Since current therapeutic approaches for PD predominantly restore dopaminergic tone to control motor symptoms, though without altering disease progression, interfering with the interaction between α-syn and lipids offers potential avenues for therapeutic strategies in PD [42].

Thus, this review focuses on two key aspects related to the reciprocal interplay between α-syn and lipids: (1) changes in lipid composition or metabolism that may impact the biochemical dynamic of α-syn aggregation and (2) alterations in the α-syn structure due to mutations or post-translational modifications (PTMs) that could influence its interaction with lipids. The final goal of this review is to elucidate this bidirectional crosstalk and how this may drive the pathological events in PD, thus offering novel targets for therapeutic interventions.

## 2. Lipids and Lipid Metabolism

**Figure 2:**
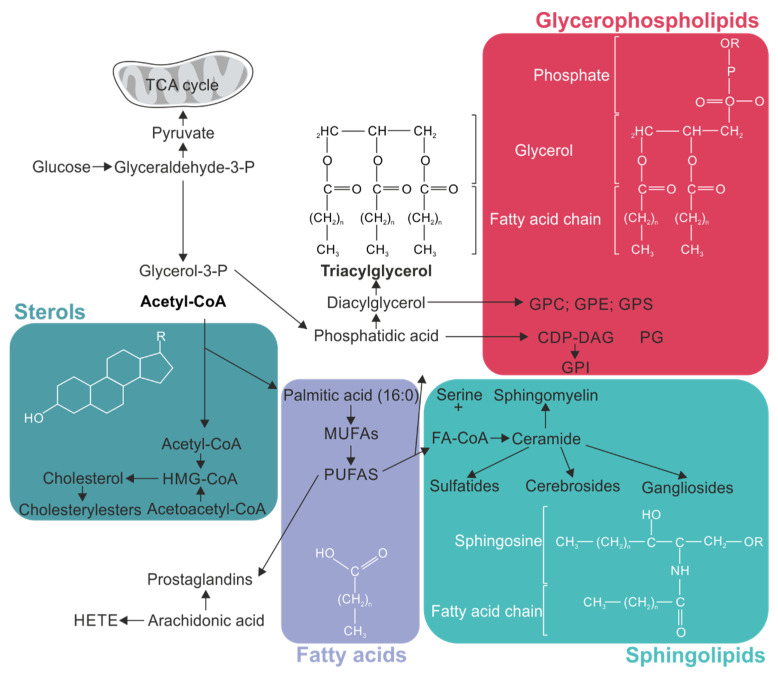
Overview of different lipid classes and their synthesis pathways. The major classes of lipids (sterols, fatty acids (FAs), sphingolipids, and glycerophospholipids) important for this review are depicted with their chemical structure and the key steps of their synthesis pathway. Eicosanoids, such as arachidonic acid, are classified as a type of FAs. CDP = cytidine diphosphate; CoA = Coenzyme A; GPC = glycerophosphocholine; GPE = glycerophosphoethanolamine; GPS = glycerophosphoserine; HETE = hydroxyeicosatetraenoic acids; HMG = β-hydroxy-β-methylglutaryl; MUFAs = monounsaturated fatty acids; P = phosphate; PUFAs = polyunsaturated fatty acids; TCA = tricarboxylic acid.

The four major classes of biomolecules in a mammalian cell are carbohydrates, proteins, nucleic acids, and lipids [43]. The latter are an essential component of the brain. Indeed, the brain has the second highest lipid content after adipose tissue in the human body, accounting 50% of the brain’s dry weight [44]. In contrast to adipose tissue, where fatty acids (FAs) are predominantly stored as triglycerides (TAG) for energy storage, the brain primarily utilizes lipids as structural components for membranes [44]. Regular biomembranes typically have a lipid:protein ratio of about 50%:50%. However, in the case of myelination in oligodendrocytes, lipids play a particularly crucial role, as myelin is characterized by an exceptionally high proportion of lipids, with a lipid:protein ratio of 70–85%:15–30% [45]. In general, lipids fulfill a broad range of roles throughout the body such as energy supply, membrane components, and precursors of vitamins and hormones. Moreover, they contribute to blood coagulation and to immune responses [46]. Lipids are taken up by nutrition or are synthesized de novo. Multiple different neural cells are able to produce its own lipids. In this review, we focus on the four major types of lipids: sterols, (including cholesterol), FAs, sphingolipids, and glycerophospholipids (Figure 2). Other lipid classes such as saccharolipids, polyketides, and prenol lipids have been reviewed elsewhere [47].

### 2.1. Lipid Metabolism in the Brain

**Figure 3:**
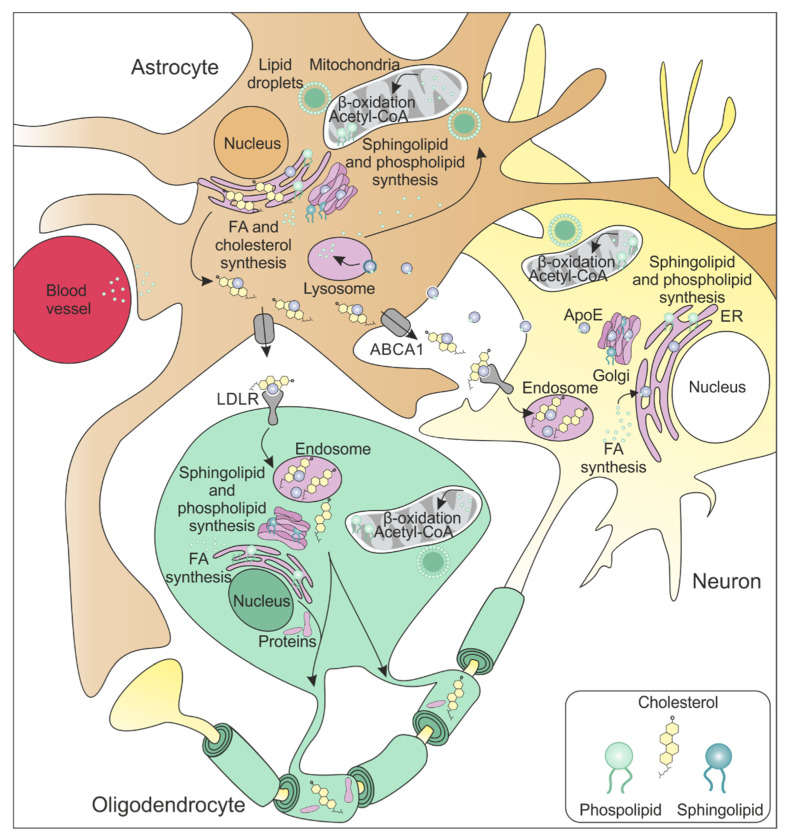
Lipid metabolism in the adult brain. All lipids are produced based on two main components: cholesterol and FAs. Cholesterol is synthesized primarily at the ER of astrocytes and is further transported to neurons and oligodendrocytes via ApoE and ABCA1 transporters. FAs, however, are produced by neurons, astrocytes, and oligodendrocytes. Additionally, FAs also bind to ApoE for their transport. FAs are used as a fuel source in β-oxidation predominantly by astrocytes, but also by neurons and oligodendrocytes. Alternatively, all neural cell types contain lipid droplets for storage. Finally, all cells are able to produce phospholipids and sphingolipids within the ER. ABCA1 = ATP-binding cassette transporter A1; ApoE = apolipoprotein E; ER = endoplasmatic reticulum; LDLR = low-density lipoprotein receptor.

All lipids except sterols, such as cholesterol, are generated from FAs [48]. While FAs pass across the blood–brain barrier [49], the entry of cholesterol into the brain is largely restricted [50]. Thus, there is a crucial need for cholesterol synthesis within the CNS [51]. In general, cholesterol can be synthesized de novo by all cells in the brain [52]. However, the neural cell type primarily responsible for cholesterol synthesis shifts from development to adulthood (Figure 3). During embryogenesis, neurons are the primary producers of cholesterol. However, during postnatal myelination, the production site shifts to oligodendrocytes, and later in adulthood, it primarily transitions to astrocytes [51,52]. Astrocytes are considered the major neural cells taking over lipid production in the brain not only of cholesterol, but also of diacylglycerol (DAG) and triacylglycerol (TAG) [53]. Thus, in the adult brain, neurons and oligodendrocytes mainly take up lipids derived from astrocytes to support synaptic function [54] and myelination [53,55]. A simplified overview of lipid metabolism in the brain is depicted in Figure 3.

#### 2.1.1. Cholesterol

The de novo cholesterol synthesis pathway is based on the acetyl-CoA pool that is converted to cholesterol in a multistep mechanism primarily in the endoplasmatic reticulum (ER) (Figure 2). The transport of cholesterol from astrocytes to neurons and oligodendrocytes is facilitated by apolipoprotein E (ApoE), also produced by astrocytes themselves [56]. Bound to ApoE, cholesterol is exported by ATP-binding cassette (ABCA1) transporters [57]. The cholesterol-ApoE complex is consequently endocytosed by low-density lipoprotein receptors (LDLR) expressed by neurons [58,59] and oligodendrocytes [60] (Figure 3). Within oligodendrocytes, cholesterol associates with the proteolipid protein (PLP) and is integrated into the myelin sheath during myelination [61,62].

#### 2.1.2. Fatty Acids

FAs are essential for various components of cell membranes and myelin, as well as for providing energy. Although FAs are able to cross the blood–brain barrier and pass through cellular membranes, neurons, oligodendrocytes, and astrocytes are able to synthesize the majority of required saturated and monounsaturated fatty acids (MUFAs) by themselves (reviewed by [63]). However, the brain and other organs severely lack the ability to produce polyunsaturated fatty acids (PUFAs) [64]. Thus, PUFAs need to be taken up by the diet [65]. FA synthesis takes place in the cytoplasm and the ER [66]. Besides serving as basis for the synthesis of complex lipids, FAs are stored as energy-rich TAGs in lipid droplets. Astrocytes are the most prominent cell type responsible for producing lipid droplets in the adult brain. However, neurons and oligodendrocytes also generate lipid droplets (as reviewed in [67]). Lipid droplets serve two important purposes: first, they help sequester free cytosolic FAs which, in the absence of lipid droplets, can be toxic to cellular structures like mitochondria by disrupting their membranes (reviewed by [68]); second, lipid droplets facilitate the transport of FAs into mitochondria, providing an essential energy supply during starvation and enabling β-oxidation [69,70]. In the brain, β-oxidation, the process of degrading FAs, is primarily observed in astrocytes, and it is also present in neurons and oligodendrocytes [71].

#### 2.1.3. Sphingolipids

Sphingolipids, including glycolipids such as gangliosides, cerebrosides, and sulfatides, require FAs for their production, particularly ceramide, which is subsequently incorporated into various complex sphingolipids, predominantly in the Golgi (reviewed by [72]).

#### 2.1.4. Phospholipids

Phospholipids, the main component of biological membranes, are produced by all the major neural cells in the brain using FAs as biochemical building blocks. The synthesis of all classes of phospholipids takes place in the ER and is initiated by two common precursors: phosphatidic acid (PA) and DAG (reviewed by [73]).

## 3. α-syn and Lipids

α-syn was originally discovered in the nucleus and the presynaptic terminals [1], where it is involved in neurotransmission and synaptic plasticity [74]. Immediately after the discovery of α-syn within Lewy bodies [75], the lipid-binding properties of α-syn were described in numerous studies where α-syn was found to associate with synaptic membranes [76]. It displays a preference for binding to negatively charged head groups of anionic lipids. This interaction is mediated by the amphipathic N-terminal region of α-syn, which is rich in lysine residues [77]. Additionally, α-syn exhibits a specific affinity to the phospholipids phosphatidylethanolamine (PE), PA, phosphatidylinositol (PI), and ganglioside due to their acidic head groups, rather than to phosphatidylserine (PS) or phosphatidylglycerol (PG) [78,79,80,81]. Moreover, α-syn contains a cholesterol-binding site (residues 67–78) [82] as well as one for glycosphingolipids (residues 34–45) [83]. It also interacts with membranes, including myelin, with a preferential interaction with membranes containing unsaturated FAs [84]. Further, α-syn preferably binds to lipid raft domains of membranes [85]. Intracellularly, α-syn also associates with mitochondrial membranes [86], although the physiological role of this interaction is still unclear.

After α-syn is bound to a membrane, it forms an α-helical structure. Membrane binding of monomeric α-syn is mediated by two steps: (1) anchoring to the membrane with the N-terminal residues 3–25, and (2) a coil-to-helix transition of residues 26–97 that are responsible for the lipid binding and act as membrane sensors [87,88,89]. Physiologically, binding of α-syn to membranes and the consecutive formation of an α-helical structure are important for soluble N-ethylmaleimide-sensitive-factor attachment receptor (SNARE)-complex assembly [14,90].

The interaction of α-syn monomers with membranes was investigated extensively; however, binding of α-syn multimers to membranes remains elusive. While monomer binding to the membrane leads to the formation of an α-helical structure, multimers do not change their secondary structure upon membrane binding [16,91]. Moreover, different α-syn multimers species with distinct structures and membrane interaction properties exist [16,92]. Nevertheless, similar to monomeric α-syn, multimers prefer binding to lipids having acidic, negatively charged head groups [78,93] and lipid raft domains of membranes [78].

There are different mechanisms underlying the interaction of α-syn and lipids in PD, which will be further elaborated in this review: (1) multiple alterations in lipid classes and metabolism have been observed in PD patients and PD models affecting the aggregation propensity of α-syn; (2) missense mutations of α-syn identified so far in familial monogenic PD are localized at the *N*-terminus, where lipid binding takes place, and indeed change its lipid-binding properties; and (3) PTMs of α-syn change its binding properties toward different forms of lipids.

### 3.1. Alterations of Lipids and Their Metabolism in PD

PD is frequently characterized as a proteinopathy; however, emerging evidence suggests that it might be described as a lipidopathy, or most likely as a combination of both (reviewed by Fanning and colleagues [94]). An analysis of three genome-wide association studies (GWAS) revealed four main biological processes relevant for PD—oxidative stress response, endosomal-lysosomal functioning, ER stress response, and immune response activation [95]. Interestingly, lipids and lipoproteins are key to all four processes [95]. Furthermore, α-syn is involved in several lipid metabolic pathways, including FA [96,97,98], TAG [99], and cholesterol metabolism [100]. Indeed, alterations in lipid metabolism have been found throughout different metabolic pathways, including FA [101,102], cholesterol [103,104], sphingolipid [105,106], and glycerophospholipid metabolism [102].

#### 3.1.1. FA Metabolism

Recently, it was demonstrated that α-syn overexpression in yeast-, rodent-, and induced pluripotent stem cell (iPSC)-derived neurons increased the formation of MUFAs, specifically oleic acid, which subsequently enhanced α-syn toxicity by altering the equilibrium of the membrane bound to soluble α-syn [107]. Coincidentally, neuronal and plasma levels of PUFAS are increased in PD patients [108,109]. Along this line, α-syn oligomerization is regulated by PUFA levels [110]. Especially docosahexaenoic acid (DHA) and α-linolenic acid (ALA) are able to bind α-syn and elevate its aggregation at low ratios, while reducing the aggregation at high ratios.

#### 3.1.2. Cholesterol Metabolism

Several oxysterols are increased in PD brains [111], and, importantly, cholesterol accumulates in lysosomes of glucocerebrosidase (*GBA*)-PD patients [112]. *GBA* mutations are associated with monogenic PD. Moreover, an increased brain cholesterol level was detected in methyl-4-phenyl-1,2,3,6-tetrahydropyridine (MPTP) mice, which is a neurotoxin-induced PD model, exacerbating dopaminergic loss in the striatum and substantia nigra. Furthermore, a high-cholesterol diet alleviated motor functions in the animals [113]. Similarly, high cholesterol levels in SH-SY5Y-differentiated neurons led to decreased dopaminergic neuronal survival [114]. Thus, selectively targeting cholesterol synthesis in the CNS might be a promising therapeutic approach.

#### 3.1.3. Sphingolipid Metabolism

Notably, several enzymes involved in sphingolipid metabolism are associated with PD. Emerging evidence implies that distinct variants within the genes for *GBA* [115] and serine palmitoyltransferase (*SPTLC1*) [116] are important risk factors for developing PD. Moreover, sphingolipids can associate with cholesterol to form lipid rafts involved in signal transduction and membrane trafficking [117], while sphingolipid metabolites such as ceramides and sphingosine-1-phosphate play important roles in cell proliferation, differentiation and apoptosis [118,119]. Thus, pharmaceutical intervention in the sphingolipid metabolic pathway might be useful for intervening in pathological processes in PD.

#### 3.1.4. Glycerophospholipid Metabolism

Farmer and colleagues observed that 17 glycerophosphocholine and lysophosphatidylcholine species were significantly reduced in the substantia nigra of 6-hydroxydopamine (6-OHDA)-lesioned PD mice using high-performance liquid chromatography coupled with mass spectrometry [120]. Specifically, the lysophosphatidylcholine species (16:0/0:0) and (18:1/0:0) were increased in this mouse model, which were also found to be increased in human fibroblasts deficient in *PARKIN*, being a risk factor for monogenic PD [121]. Both lysophosphatidylcholine species contribute to inflammatory signaling in the pathogenesis of PD [122]. Moreover, the *PARKIN*-mutant fibroblasts exhibit higher levels of glycerophosphoserine, glycerophosphoinositol, and gangliosides GM2 and GM3 [121]. Elevated levels of glycerophosphoinositol and glycerophosphoserine may cause defects in mitochondrial turnover [121]. Additionally, PE was found to be reduced in the substantia nigra and midbrain of PD patients [123,124]. In yeast cells, PE deficiency has been linked to the disturbance of α-syn homeostasis, highlighting a potential functional role in the pathology of PD [125]. To produce glycerophospholipids, DAGs are needed. Moreover, DAG is able to act as second messenger in nuclear lipid signaling. Using liquid chromatography–mass spectrometry, Wood and colleagues identified increased levels of DAGs, with both monounsaturated and polyunsaturated hydrocarbon chains, in the frontal cortex of PD patients correlating with the severity of neuropathology [126]. Moreover, they observed a significant decrease in the levels of PA 16:0 in PD patients [126].

### 3.2. Effects of Missense Mutations on the Binding Capacity of α-syn to Lipids

While membrane binding of α-syn may be important for its physiological function, abnormal alterations of α-syn such as overexpression, aggregation, or mutation may have pathological effects upon membrane binding. For example, association of abnormal α-syn with mitochondrial membranes has detrimental effects [127,128]. In dopaminergic and primary neurons overexpressing α-syn, mitochondrial impairment associated with an increase in oxidative stress and reduced cell viability was observed [128,129]. Typically, α-syn binds to membranes with its first 25 amino acid residues at the N-terminus when the lipid-to-protein ratio is high. However, a reduction in the lipid-to-protein ratio causes α-syn to interact with the membrane by binding with the first 97 amino acid residues [130]. Thus, the N-terminal domain of the α-syn gene, where missense mutations identified so far in familial PD have been found, is of special interest.

Missense mutations within the N-terminal region of the *SNCA* gene have detrimental effects on the binding capacity of α-syn to lipids. Here, we present a summary of the impact of point mutations in the *SNCA* gene (V15A, A18T, A29S, A30P, E46K, H50Q, G51D, A53E, A53T, A53V) associated with monogenic PD, focusing on their effects on α-syn aggregation and, in particular, their interaction with lipids (Table 1). The effect of the mutations on the aggregation of α-syn is reviewed more comprehensively elsewhere [131].

A recently discovered V15A mutation led to alterations in the binding capacity of α-syn to lipids in vitro [132]. V15A-mutated α-syn showed a decreased affinity to phospholipids accompanied by an increased aggregation property and seeding activity compared to wildtype α-syn [132].

A18T and A29S are less toxic than wildtype α-syn in yeast [133]. Moreover, yeast strains with altered triglycerides reduce α-syn toxicity in both variants [133].

A reduced binding to membrane lipids was observed in the A30P variant in vitro [134] and in vivo [135]. Fibril formation was slower compared to wildtype α-syn in the A30P variant [136]. Interestingly, lipid raft association is required for the synaptic localization of α-syn, and the interaction of α-syn with lipid rafts is hindered by the A30P mutation [85].

It is additionally noteworthy that alterations at the N-terminal glutamate residues appear to exert a significant influence on the interaction between α-syn and lipids. Several studies have demonstrated that three glutamate-to-lysine mutations, namely, the pathogenic mutation E46K and two additional artificial mutations, E35K and E61K, in different combinations (“1K”: E46K; “2K”: E35K, E46K; and “3K”: E35K, E46K, E61K) enhance lipid interactions and disrupt membrane selectivity [137,138]. In these studies, the lipid-binding and lipid-remodeling abilities of “3K” were characterized. Nuber and colleagues first observed that E46K mutants increase N-to-C interactions and coil compactness in the structure of the lipid-unbound protein. Moreover, the conformation of α-syn was also affected upon interaction with a curved lipid bilayer in the E46K-like mutants. Interestingly, glutamate-to-lysine mutations mildly increased the affinity for curved membranes and caused a progressive loss of curvature selectivity [137].

The H50Q mutation enhances α-syn aggregation and toxicity without affecting the binding capacity to membranes in vitro [139,143].

In the G51D variant, a reduced binding to membrane lipids was detected in vitro [134] and in vivo [135]. Furthermore, the formation of metal ion-induced pathologic oligomers was increased, and fibril formation was accelerated in this variant [136].

While fibril formation was increased in the A53T variant [136], the binding capacity of α-syn to membranes was not changed [141]. Perissinotto and colleagues analyzed the interaction of A53T α-syn specifically with artificial lipid bilayers mimicking lipid rafts [142]. They demonstrated that distinct monomeric and multimeric α-syn species interact differently with the artificial lipid rafts. The α-syn monomers caused membrane thinning, while iron-mediated oligomers did not impair the membrane. In both aggregation states, the A53T variant facilitated the interaction with membrane lipids [142].

Furthermore, recent studies have shed light on a potential impact of α-syn mutation on retromer-mediated endosomal trafficking. The proposition arises from the identification of mutations in the retromer gene VPS35, known to cause late-onset PD [144]. Retromer is a multi-subunit protein complex coating the cytosolic site of early endosomes, and it plays a pivotal role in endosomal trafficking and sorting [145]. Notably, a recent yeast model study provided mechanistic insights by revealing that the A53T α-syn mutation specifically reduces retromer-mediated trafficking of the conserved membrane-bound proprotein convertase Kex 2 [146]. This disruption might be caused by alterations in the binding ability of the A53T α-syn to the anionic phospholipid phosphatidylinositol 3′-phosphate (PI3P) in the endosomal membrane [146].

Surface plasmon resonance spectroscopy suggests that the A53V and A53E variants exhibit a low binding affinity to membranes compared to wildtype [140]. This low membrane binding capacity may be due to the nonpolar nature of valine which does not interact with the negatively charged membrane surface [140].

### 3.3. Binding Capacity of Posttranslational Modified α-syn to Lipids

Numerous studies have demonstrated that the interaction between α-syn and membranes is modified by PTMs. Despite its small size, α-syn undergoes a variety of PTMs including phosphorylation, acetylation, nitration, ubiquitination, truncation, glycosylation, and glycation (reviewed by [147]) (Figure 1). PTMs regulate the physiological function of α-syn but may also be linked to the pathogenic potential of the protein. Specifically, PTMs significantly influence the structure and aggregation propensity of α-syn as well as its interactions with lipids. The effects of PTMs on protein aggregation and toxicity have been extensively reviewed elsewhere [148,149,150,151]. Here, we in particular address the impact of PTMs on α-syn-lipid interactions. An overview of the detailed effects of PTMs on α-syn-lipid interactions is depicted in Table 2.

#### 3.3.1. Phosphorylation

Phosphorylation is mediated by kinases [168] and reversed by phosphatases, respectively [169]. Phosphorylation is an esterification reaction involving the attachment of a phosphoryl group to the hydroxyl group of the side-chains of specific amino acids such as serine, tyrosine, and threonine [170]. α-syn is most commonly phosphorylated on serine [153,171] and tyrosine residues [172,173,174,175]. In particular, phosphorylated α-syn at S87 [153] and S129 [171] is enriched in Lewy bodies [176]. S129 is even enriched by 90% [176]. The current literature presents divergent findings concerning the adverse and beneficial effects of phosphorylation on the interaction of α-syn with lipids. Phosphorylation on S87 and S129 was shown to alter the conformation of membrane-bound α-syn by destabilizing the α-helical conformation, leading to a decreased affinity to lipid vesicles [153,154,155]. However, conflicting results from Samuel and colleagues demonstrated no difference in membrane binding to synaptosomes upon phosphorylation at S129 [156].

#### 3.3.2. Acetylation

Acetylation of α-syn is mediated by irreversible addition of an acetyl group to the amine group of the N-terminus (methionine) by histone acetyl transferase, resulting in a decreased positive charge [177]. It has been estimated that over 80% of α-syn molecules are acetylated [171]. Interestingly, acetylated α-syn is found to be enriched in Lewy bodies and affected brain regions from PD patients [171,178]. N-terminal acetylation induced α-helical structures of monomeric, soluble α-syn and thereby decreased its aggregation rates [179]. Due to the decreased positive charge upon acetylation, binding to negatively charged phospholipid head groups is influenced in a way that the affinity of α-syn for membrane binding is enhanced [157], while its structural properties were not altered [157]. In addition, N-terminal acetylated α-syn localizes to highly curved, ordered membranes with a preference for lipid rafts under cell-free conditions [180]. The effect of site-specific acetylation as well as its neurotoxic potential need to be further investigated.

#### 3.3.3. Nitration

Nitration is an irreversible aversive PTM that occurs on tyrosine residues, in particular in the presence of oxidative stress [181]. This PTM has been associated with several neurodegenerative diseases, including PD [181,182]. Nitrated α-syn was not only enriched in Lewy bodies [181,183], but also led to increased oligomerization of α-syn [184] as well as cytotoxicity in cells [185] and in the substantia nigra of rats [186]. Furthermore, α-syn nitration induced a reduced formation of α-helical structures and a decreased binding affinity of α-syn to negatively charged lipid vesicles [158]. Specifically, after nitration at Y39 or Y125, α-helix formation upon α-syn binding to lipid vesicles was diminished, and fibrils showed a distinct morphology compared to wildtype α-syn [158]. Moreover, nitration of Y125, Y133, and Y136 interfered with the binding affinity of α-syn to lipid vesicles [158].

#### 3.3.4. Ubiquitination

Ubiquitination is a reversible PTM important for intracellular protein homeostasis. This type of PTM involves the attachment of ubiquitin, a small regulatory protein consisting of 76 amino acids, to lysine residues of a target protein through an isopeptide bond. This process is essential for targeted degradation and is mediated by three enzymes: the ubiquitin-activating enzyme E1, the ubiquitin-conjugating enzyme E2, and the ubiquitin ligase E3 [187]. α-syn contains nine lysine residues potentially being ubiquitinated (K6, K10, K12, K21, K23, K32, K34, K43, and K96). Ubiquitinated α-syn is present in Lewy bodies [188] and promotes fibril formation to a different degree depending on the position of ubiquitination in α-syn [189]. In detail, ubiquitination at K10 and K23 displayed similar fibril levels with altered kinetics of formation compared to wildtype α-syn, while K6, K12, and K21 slightly reduced fibril formation, and K32, K34, K43, and K96 reduced fibril formation more severely [189]. Moreover, it was demonstrated that ubiquitination at K6, K23, K43, and K96 had no effect on the α-helical secondary structure of α-syn after binding to lipid vesicles [159,160]. Alterations of the lipid-binding properties of α-syn upon ubiquitination are still largely unknown. It was suggested that ubiquitination of lysine residues within the N-terminal KTKEGV repeat motifs may prevent membrane binding of α-syn [190].

#### 3.3.5. Truncation

Truncation of proteins occurs due to a dysfunctional protein homeostasis machinery leading to incomplete metabolization of α-syn by a number of enzymes, such as plasmin [191], neurosin [192], cathepsin D [193], caspase 1 [194], calpain 1 [195], and other proteinases [196]. α-syn is irreversibly truncated at the N- or C-terminus and present in over 15% of α-syn in Lewy bodies [197]. An overview of the possible truncations of α-syn is reviewed by Sorrentino and colleagues [198]. Notably, N-terminally truncated α-syn variants, 5–140, 39–140, 65–140, 66–140, 68–140, and 71–140, and C-terminally truncated α-syn variants, 1–101. 1–103, 1–115, 1–122, 1–124, 1–135, and 1–139 have been detected in different brain regions of PD patients so far [171,197,199]. Since the N-terminal domain of α-syn determines its lipid-binding capacity, truncation within this site may reduce physiological membrane binding. In general, truncation of α-syn is able to induce aggregation and toxicity in vitro [200] and in vivo [201] by increasing the spread of α-syn through synaptically coupled neuroanatomical tracts [202]. The impact of C-terminal truncations of α-syn on the aggregation of the protein was investigated in more detail, since oligomerization and aggregation of α-syn is mediated mainly by the C-terminus [162]. In any case, C-terminal truncation of α-syn reduces its solubility and affects its membrane-binding properties as well leading to site-specific neurotoxic effects [161,162,163,164,165].

#### 3.3.6. Glycosylation

Glycosylation is a type of reversible enzyme-dependent PTM, in which N-acetylglucosamine (GlcNAc), an amide derivative of glucose, is transferred from uridine diphosphate-GlcNAc (UDP-GlcNAc) to the hydroxyl group of threonine or serine residues of a protein [203]. The addition of GlcNAc is catalyzed by O-GlcNAc-transferase, while its removal is mediated by O-GlcNAcase [203]. So far, nine residues of α-syn (T33, T44, T54, T59, T64, T72, T75, T81, and S87) have been reported as potential sites of glycosylation [204,205,206]. In general, glycosylation of α-syn at various position reduces aggregation and toxicity of α-syn [207,208], though it does not affect binding of α-syn to phospholipid membranes [166]. Interestingly, triple glycosylation at T72, T75, and T81 inhibited theα-helical structure of α-syn upon membrane binding [166]. Notably, glycosylation has an impact on other PTMs. In particular, glycosylation of α-yn was shown to prevent its phosphorylation at S129, whereas it promotes phosphorylation at S87 [207]. No aversive effects have been reported so far for glycosylation of α-syn.

#### 3.3.7. Glycation

Glycation of α-syn is based upon a non-enzymatic reaction of its lysine residues with reactive carbonyl species as a side product of glycolysis, known as glycation [209]. One of the most prevalent end products of glycation is Nε-(carboxyethyl)lysine (CEL) [210]. It was demonstrated that glycation potentiates α-syn-associated neurodegeneration in PD [211]. Recently, it was observed that CEL formation on α-syn reduces its binding affinity towards sodium dodecyl sulfate (SDS) micelles used as a membrane mimic without affecting the α-helical structure of α-syn [167]. In PD, glycation of α-syn is implicated in protein aggregation and Lewy body formation, while site-specific effects are still poorly understood.

## 4. Therapeutic Potential

**Figure 4:**
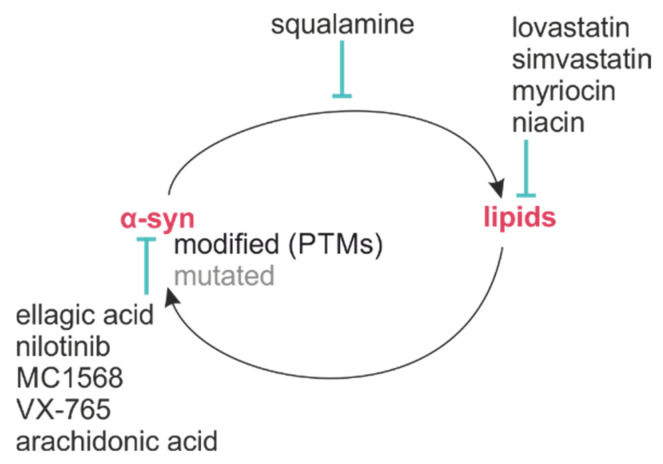
Bidirectional interaction of α-syn and lipids. Modifications of α-syn via mutation or PTMs can alter lipid-binding properties, while changes in lipid composition or metabolism alter pathological properties of α-syn. Moreover, different therapeutics may be used to modulate α-syn and lipids within the CNS, or the interaction between both.

Due to the importance of the interaction of α-syn with lipids, strategies are emerging to modulate this interaction. Several therapeutic approaches have already been investigated and are currently being tested in multiple clinical trials (Table 3). A schematic of the bidirectional interaction of α-syn and lipids and the possible therapeutic interventions is depicted in Figure 4.

### 4.1. Enzymes Involved in Lipid Metabolism

One potential approach is targeting activities of proteins involved in lipid metabolism, including enzymes and lipid transporters. For example, Vincent and colleagues were able to ameliorate α-syn-induced cytotoxicity by inhibiting the highly conserved enzyme stearoyl-CoA desaturase in iPSC-derived neuronal models [228]. This enzyme catalyzes the rate-limiting step in the formation of MUFAs; thus, inhibition of this enzyme reduces the levels of unsaturated membrane lipids [229]. Moreover, inhibition of stearoyl-CoA desaturase was able to reduce the formation of α-syn inclusions in the “3K” variant of the E46K mutation [230]. This result was confirmed by Nuber and colleagues in cultured human neurons, in “3K” neural cultures, and “3K” α-syn mice [42].

Along this line, inhibition of the key enzymes within the cholesterol biosynthesis pathway induced accumulation of 8, 9-unsaturated sterols driving oligodendrocyte formation and remyelination [231]. For example, Lovastatin reduces cholesterol levels by inhibiting HMG-CoA reductase, which catalyzes the rate-limiting step in the cholesterol synthesis pathway [232]. Thereby, Lovastatin reduces α-syn accumulation and its phosphorylation in vitro in HEK293 cells, SH-SY5Y cells, and in primary human neurons [212,213] and in vivo in different transgenic mouse models that neuronally overexpress human α-syn [214]. Similarly, Simvastatin or other statins can be used as therapeutic approaches [212].

Furthermore, the inhibition of the de novo ceramide synthesis by myriocin, an inhibitor of serine palmitoyltransferase, reduced oxidative stress and inflammation and increased vesicular trafficking in SH-SY5Y cells treated with α-syn fibrils [217].

In summary, these observations suggest that inhibition of important enzymes participating in lipid metabolism may be able to prevent α-syn-mediated toxicity. Based on this evidence, development of inhibitors specifically targeting these enzymes is emerging as potential therapeutic strategy for PD and other synucleinopathies.

### 4.2. Membrane Binding of α-syn

Another possibility is to modulate binding of altered α-syn to membranes, for example, by using competitive compounds. It has been described that polyphenolic compounds compete effectively with α-syn for membrane binding and are thus considered a strong potential therapeutic candidate for PD and other synucleinopathies [219]. One polyphenolic compound that has an inhibitory effect toward α-syn oligomerization and fibrillation in vitro is ellagic acid [218]. Hence, α-syn aggregation was reduced, and cell survival increased [218]. Another molecule is squalamine [219], an antimicrobial aminosterol originally discovered in 1993 in the dogfish shark, *Squalus acanthias* [233]. Indeed, squalamine carries a net positive charge and shows a high binding affinity for anionic phospholipids [234]. By competing with α-syn for binding to the membranes, squalamine specifically inhibits the initiation of the aggregation process of α-syn [219]. Thus, it alleviates the toxicity of α-syn oligomers in neuronal cells and in a *Caenorhabditis elegans* model of PD [219].

### 4.3. PTMs

Since PTMs modify the interaction of α-syn with lipids, interfering with PTM pathways is considered as a novel therapeutic target for PD. Modulation of phosphorylation of α-syn is achieved by pharmacological modulation of kinases and phosphatases [235,236,237,238]. For example, using nilotinib, a Food and Drug Administration (FDA)-approved cancer treatment, to inhibit the kinase c-Abl leads to reduced phosphorylation, enhanced clearance of α-syn, reduced neurotoxicity, and improved motor behavior in a mouse model of PD [220].

Another possibility is to enhance the phosphatase activity of phosphoprotein phosphatase 2A (PP2A) by increasing methylation of the enzyme to decrease α-syn phosphorylation at S129, leading to decreased α-syn aggregation and toxicity in mice [239].

To target ubiquitination, antibodies inhibiting the ubiquitin E3 ligase were developed, which decreased the expression and aggregation of α-syn and improved cell viability in vitro [240].

Pharmacological inhibition of class IIa histone deacetylases (HDACs), which are important enzymes for the modulation of α-syn by acetylation, using MC1568 increased neurite density and cell survival and protected against the neurotoxin-treated SY5Y cells [221]. However, effects on the binding capacity of α-syn to lipids are not yet known.

Truncations of α-syn may be reduced by immunotherapy or pharmacological inhibition of caspases [222,241]. So far, therapeutic approaches concentrate on C-terminal truncations. One example is VX-765 that inhibits caspase-1, which cleaves α-syn at D121, thereby improving motor symptoms, neurodegeneration, and neuroinflammation in a transgenic mouse model of MSA [222].

Since glycosylation of α-syn reduces α-syn aggregation, pharmacological inhibition of O-GlcNAcase increases the glycosylation levels of α-syn, resulting in a lower aggregation of α-syn [242]. Moreover, glycosylation inhibits calpain-mediated C-terminal α-syn truncations, thus reducing aggregation of α-syn as well. Similarly, glycosylation competes with phosphorylation in targeting hydroxyl groups on serine and threonine residues, thereby protecting α-syn from increased aggregation caused by phosphorylation [207]. Along this line, accumulation of S129 α-syn was reduced in the substantia nigra in an adeno-associated virus-generated A53T mouse model of PD by pharmacological inhibition of O-GlcNAcase [243].

### 4.4. Neuroprotective Lipids

Given the neuroprotective effects of some lipids, their direct administration is emerging as a promising therapeutic strategy to alleviate α-syn-mediated cytotoxicity. One example is arachidonic acid, an essential FA that induces the formation of ordered, α-helical structured α-syn multimers being resistant to fibrillation [223]. Another target are PUFAs, especially omega-3, an important component of membranes (reviewed by [244]). Among other positive effects on PD, omega-3 PUFAs inhibit neuroinflammation, maintain α-syn degradation, and improve membrane fluidity (reviewed by [244]), thus emerging as a therapeutic strategy. Another potential nutrient is niacin/nicotinamide, a precursor of NADH and cofactor of mitochondrial enzymes [245,246]. Nicotinamide has already been linked to neuroprotection in PD and has shown to protect against MPTP induced neurotoxicity in mice [224,225]. Furthermore, nicotinamide prevented mitochondrial dysfunction in a cellular model and improved motor behavior in a *Drosophila* model of PD [226].

### 4.5. Environmental Factors

Since a variety of environmental factors affect lipid homeostasis, targeting these factors is a promising approach. Dietary nutrients are the main substrates of the gut microbiota and can have an impact on the composition and metabolic activity of these microbiota (reviewed by [247]). These processes lead to the production of intermediate metabolites affecting host energy homeostasis, glucose, and lipid metabolism [248]. For example, nutrition rich in antioxidants might be neuroprotective in PD [249]. Since increased lipid droplet formation in dopaminergic neurons has been correlated with iron accumulation, pharmacological administration of iron chelators such as deferiprone arises as a therapeutic strategy. Deferiprone reduces iron depositions in the substantia nigra accompanied by alleviated motor deficits in an initial clinical trial in early PD [227]. However, it could not be confirmed lately.

Overall, lipids and their metabolism in the CNS contribute profoundly to the identification of novel therapeutic interventions for PD.

## 5. Conclusions

α-syn has been associated with PD and other synucleinopathies for over two decades. However, this discovery has not yet led to the development of effective and causative therapeutic approaches. Thus, this review focuses on an important aspect of α-syn, namely its interaction with lipids in the CNS. On the one hand, alterations of lipids and different metabolic pathways influence the function and the dysfunction of this protein. On the other hand, the interference of α-syn with lipids is changed in PD due to different factors, such as point mutations within the lipid-binding region (Table 1) or PTMs (Table 2). Focusing on PTMs, researchers have identified compounds that modulate PTMs, which reduce the aggregation of α-syn and modify its binding properties to membranes. Moreover, targeting enzymes involved in various stages of lipid metabolism and exploring the neuroprotective potential of certain lipids have emerged as promising therapeutic avenues. Efforts toward a more detailed characterization of α-syn interventions in lipid metabolism and function will lead to a more in-depth assessment of the protein’s implications for therapeutic purposes. In conclusion, investigation of the bidirectional interaction of α-syn with lipids is advancing our comprehension of the pathology in PD and other synucleinopathies, suggesting that these disorders are not solely a consequence of protein pathology but also influenced by lipid-related processes. Thus, PD is not simply a synucleinopathy but rather a meta-disease composed of several different aspects.

## Figures and Tables

**Table 1:** Summary of PD-related point mutations of α-syn and their effects on its binding capacity to membranes.

Mutation	Effects on Lipid Membranes	Ref.
V15A	decreased affinity to phospholipids accompanied by an increased aggregation and seeding activity	[132]
A18T	less toxic than wildtype α-syn altered triglycerides reduce α-syn toxicity	[133]
A29S	less toxic than wildtype α-syn altered triglycerides decrease α-syn toxicity enhanced acetylation or SUMOylation are protective against α-syn toxicity	[133]
A30P	reduced binding to membranes formation of metal ion-induced pathologic oligomers was increased fibril formation is slower in A30P mutants compared to wildtype interaction of α-syn with lipid rafts is hindered	[85,134,135,136]
E46K	increased lipid interactions and disrupted membrane selectivity increased N-to-C interactions and coil compactness in the structure of lipid-unbound α-syn conformation of α-syn is altered upon interaction with a curved lipid bilayer	[137,138]
H50Q	enhances α-syn aggregation and toxicity without affecting the binding capacity to lipid membranes	[139]
G51D	decreased binding to lipid membrane fibril formation was accelerated	[136]
A53E	α-syn exhibits a low lipid binding capacity compared to wildtype	[140]
A53T	does not change the binding capacity of α-syn to membranes formation of metal ion-induced pathologic oligomers and fibril formation are increased α-syn monomers cause membrane thinning and facilitate the interaction with artificial lipid rafts iron-mediated oligomers do not impair the membrane, but facilitate the interaction with artificial lipid rafts no effect on the interaction of α-syn with lipid rafts	[85,136,141,142]
A53V	low binding affinity to membranes compared to wildtype less toxic than wildtype α-syn altered triglycerides reduce α-syn toxicity enhanced acetylation or SUMOylation are protective against α-syn toxicity	[133,140]

**Table 2:** Summary of PD-related point mutations of α-syn and their effects on its binding to membranes.

PTM	Position	Effects on Membranes	Ref.
Phosphorylation	Y39	diminished lipid binding of α-syn and increased axonal pathology in transgenic PD mice	[152]
	S87	conformational change in membrane-bound α-syn decreased affinity to lipid vesicles reduced aggregation of α-syn	[153]
	S129	reduced binding of α-syn monomers and Fe^3+^-induced oligomers to lipid vesicles fewer α-helical structures, decreased binding, and disruption of lipid vesicles no difference in membrane binding to synaptosomes in the A30P variant, α-syn membrane binding was increased, leading to disruption of membranes in the A53T variant, binding to membranes was reduced	[154,155,156]
Acetylation	M1	increased affinity of α-syn to membrane binding without structural alterations	[157]
Nitration	Y39	less α-helical structure formation upon lipid vesicle binding disrupted binding affinity of α-syn to membranes	[158]
	Y125	less α-helical structure formation upon lipid vesicle binding disrupted binding affinity of α-syn to membranes	
	Y133, Y136	disrupted binding affinity of α-syn to lipid vesicles	
Ubiquitination	K6, K23, K43, K96	no alterations in secondary structure of α-syn upon lipid binding	[159,160]
Truncation	1–100	less potential inducing curvature upon membrane binding compared to full-length protein	[161]
	1–103	produces mature fibrils in the presence of phospholipid vesicles	[162]
	1–115	upon lipid binding, 1–115 truncated α-syn shows higher α-helical levels compared to full-length α-syn facilitating lipid binding	[163]
	1–119	aggregates faster than full-length α-syn in the presence of phospholipid vesicles	[162]
	1–120	reduced α-syn fibrillation and increased lipid binding predisposition upon methylphenidate treatment	[164]
	1–121	similar aggregation to full-length α-syn in the presence of phospholipid vesicles decreased ability to distort phospholipid membranes higher toxicity compared to full-length α-syn	[165]
Glycosylation	T72	reduction in fibril formation, aggregation, and toxicity of monomeric α-syn in vitro, while binding affinity to lipid vesicles was unaltered	[166]
	T75	reduction in fibril formation, aggregation, and toxicity of monomeric α-syn in vitro, while binding affinity to lipid vesicles was unaltered	
	T81	reduction in fibril formation, aggregation, and toxicity of monomeric α-syn in vitro, while binding affinity to lipid vesicles was unaltered	
	S87	reduction in fibril formation, aggregation, and toxicity of monomeric α-syn in vitro, while binding affinity to lipid vesicles was unaltered	
	T72, T75, and T81	inhibited the α-helical structure of α-syn upon membrane binding	
Glycation	Lysine	reduced binding affinity towards sodium dodecyl sulfate (SDS) micelles without affecting the α-helical structure of α-syn disruption of lipid vesicles upon α-syn binding	[167]

**Table 3:** Summary of current therapeutic approaches investigated experimentally or in clinical trials. https://www.clinicaltrials.gov, accessed on 3 August 2023.

Compound	Target	Effect	Clinical Trial	Clinical Trial PD	Ref.
Lovastatin	HMG-CoA reductase	reduces α-syn accumulation and its phosphorylation in vitro in HEK293 cells, SH-SY5Y cells, and in primary human neurons and in vivo in different transgenic mouse models that neuronally overexpress human α-syn	rheumatoid arthritis, cancer, etc.	Phase II	[212,213,214]
Simvastatin	HMG-CoA reductase	prevents MPTP-induced striatal dopamine depletion and protein tyrosine nitration in mice, and protects dopaminergic neurons in the substantia nigra, attenuates the expression of proinflammatory molecules, and improves motor deficits in the MPTP model of PD	hyper-lipidemia, diabetes, MS, etc.	Phase II	[212,215,216]
Myriocin	de novo ceramide synthesis	reduced oxidative stress and inflammation and increased vesicular trafficking in SH-SY5Y cells treated with α-syn fibrils	no	no	[217]
Ellagic acid	α-syn	polyphenolic compound that has an inhibitory effect toward oligomerization and fibrillation of α-syn in vitro, reduces α-syn aggregation, and increases cell survival	prostate cancer phase III	no	[218]
Squalamine	competitive of α-syn	specifically inhibits the initiation of aggregation of α-syn and alleviates its toxicity in neuronal cells and in a *Caenorhabditis elegans* model of PD	macular degeneration phase II and III	no	[219]
Nilotinib	α-syn kinase c-Abl	enhanced clearance of α-syn, reduced neurotoxicity, and improved motor behavior in a mouse model of PD	AD phase 3, leukemia, etc.	no	[220]
MC1568	class IIa histone deacetylases	increased neurite density and cell survival and protected against the neurotoxin-treated SY5Y cells	cancer	no	[221]
VX-765	caspase-1	reduces neurodegeneration, motor symptoms, and neuroinflammation in a mouse model of MSA	no	no	[222]
Arachidonic acid	α-syn	essential FA that induces the formation of ordered, α-helical structured α-syn multimers being resistant to fibrillation	autism, fibrosis, diabetes, etc.	no	[223]
Niacin/Nicotin-amide	Poly (ADP-ribose) polymerase	precursor of NADH and cofactor of mitochondrial enzymes that protects from MPTP-induced neurotoxicity in mice and prevents mitochondrial dysfunction in a cellular model and improves motor behavior in a *Drosophila* model of PD	hyperlipidemia, myopathy, etc.	interventional study	[224,225,226]
Deferiprone	ferric ions	iron chelator that reduces iron depositions in the substantia nigra accompanied by alleviated motor deficits in a clinical trial in early PD	HIV, ALS, heart disease, etc.	failed	[227]

## Data Availability

Not applicable.

## References

[1] Maroteaux L., Campanelli J.T., Scheller R.H. (1988). Synuclein: A neuron-specific protein localized to the nucleus and presynaptic nerve terminal. J. Neurosci..

[2] Iwai A., Masliah E., Yoshimoto M., Ge N., Flanagan L., De Silva H.R., Kittel A., Saitoh T. (1995). The precursor protein of non-Aβ component of Alzheimer’s disease amyloid is a presynaptic protein of the central nervous system. Neuron.

[3] Gardai S.J., Mao W., Schüle B., Babcock M., Schoebel S., Lorenzana C., Alexander J., Kim S., Glick H., Hilton K. (2013). Elevated alpha-synuclein impairs innate immune cell function and provides a potential peripheral biomarker for Parkinson’s disease. PLoS ONE.

[4] Badawy S.M.M., Okada T., Kajimoto T., Hirase M., Matovelo S.A., Nakamura S., Yoshida D., Ijuin T., Nakamura S.-I. (2018). Extracellular α-synuclein drives sphingosine 1-phosphate receptor subtype 1 out of lipid rafts, leading to impaired inhibitory G-protein signaling. J. Biol. Chem..

[5] Bellani S., Sousa V.L., Ronzitti G., Valtorta F., Meldolesi J., Chieregatti E. (2010). The regulation of synaptic function by α-synuclein. Commun. Integr. Biol..

[6] Di Rosa G., Puzzo D., Sant Angelo A., Trinchese F., Arancio O. (2003). Alpha-synuclein: Between synaptic function and dysfunction. Histol. Histopathol..

[7] Cheng F., Vivacqua G., Yu S. (2011). The role of alpha-synuclein in neurotransmission and synaptic plasticity. J. Chem. Neuroanat..

[8] Baptista M.J., O’Farrell C., Daya S., Ahmad R., Miller D.W., Hardy J., Farrer M.J., Cookson M.R. (2003). Co-ordinate transcriptional regulation of dopamine synthesis genes by α-synuclein in human neuroblastoma cell lines. J. Neurochem..

[9] Fauvet B., Mbefo M.K., Fares M.-B., Desobry C., Michael S., Ardah M.T., Tsika E., Coune P., Prudent M., Lion N. (2012). α-Synuclein in central nervous system and from erythrocytes, mammalian cells, and Escherichia coli exists predominantly as disordered monomer. J. Biol. Chem..

[10] Weinreb P.H., Zhen W., Poon A.W., Conway K.A., Lansbury P.T. (1996). NACP, a protein implicated in Alzheimer’s disease and learning, is natively unfolded. Biochemistry.

[11] Dettmer U., Newman A.J., Soldner F., Luth E.S., Kim N.C., von Saucken V.E., Sanderson J.B., Jaenisch R., Bartels T., Selkoe D. (2015). Parkinson-causing alpha-synuclein missense mutations shift native tetramers to monomers as a mechanism for disease initiation. Nat. Commun..

[12] Bartels T., Choi J.G., Selkoe D.J. (2011). α-Synuclein occurs physiologically as a helically folded tetramer that resists aggregation. Nature.

[13] Wang W., Perovic I., Chittuluru J., Kaganovich A., Nguyen L.T., Liao J., Auclair J.R., Johnson D., Landeru A., Simorellis A.K. (2011). A soluble α-synuclein construct forms a dynamic tetramer. Proc. Natl. Acad. Sci. USA.

[14] Burre J., Sharma M., Sudhof T.C. (2014). alpha-Synuclein assembles into higher-order multimers upon membrane binding to promote SNARE complex formation. Proc. Natl. Acad. Sci. USA.

[15] Chen S.W., Drakulic S., Deas E., Ouberai M., Aprile F.A., Arranz R., Ness S., Roodveldt C., Guilliams T., De-Genst E.J. (2015). Structural characterization of toxic oligomers that are kinetically trapped during alpha-synuclein fibril formation. Proc. Natl. Acad. Sci. USA.

[16] Fusco G., Chen S.W., Williamson P.T., Cascella R., Perni M., Jarvis J.A., Cecchi C., Vendruscolo M., Chiti F., Cremades N. (2017). Structural basis of membrane disruption and cellular toxicity by α-synuclein oligomers. Science.

[17] Volpicelli-Daley L.A., Luk K.C., Patel T.P., Tanik S.A., Riddle D.M., Stieber A., Meaney D.F., Trojanowski J.Q., Lee V.M. (2011). Exogenous alpha-synuclein fibrils induce Lewy body pathology leading to synaptic dysfunction and neuron death. Neuron.

[18] Osterberg V.R., Spinelli K.J., Weston L.J., Luk K.C., Woltjer R.L., Unni V.K. (2015). Progressive aggregation of alpha-synuclein and selective degeneration of lewy inclusion-bearing neurons in a mouse model of parkinsonism. Cell. Rep..

[19] Coon E.A., Singer W. (2020). Synucleinopathies. Continuum.

[20] Parkinson J. (1969). An essay on the shaking palsy. Arch. Neurol..

[21] National Collaborating Centre for Chronic Conditions (2006). Parkinson’s Disease: National Clinical Guideline for Diagnosis and Management in Primary and Secondary Care.

[22] Polymeropoulos M.H., Lavedan C., Leroy E., Ide S.E., Dehejia A., Dutra A., Pike B., Root H., Rubenstein J., Boyer R. (1997). Mutation in the α-synuclein gene identified in families with Parkinson’s disease. Science.

[23] Damier P., Hirsch E., Agid Y., Graybiel A. (1999). The substantia nigra of the human brain: II. Patterns of loss of dopamine-containing neurons in Parkinson’s disease. Brain.

[24] Krüger R., Kuhn W., Müller T., Woitalla D., Graeber M., Kösel S., Przuntek H., Epplen J.T., Schols L., Riess O. (1998). AlaSOPro mutation in the gene encoding α-synuclein in Parkinson’s disease. Nat. Genet.

[25] Zarranz J.J., Alegre J., Gómez-Esteban J.C., Lezcano E., Ros R., Ampuero I., Vidal L., Hoenicka J., Rodriguez O., Atarés B. (2004). The new mutation, E46K, of α-synuclein causes parkinson and Lewy body dementia. Ann. Neurol. Off. J. Am. Neurol. Assoc. Child Neurol. Soc..

[26] George J.M., Jin H., Woods W.S., Clayton D.F. (1995). Characterization of a novel protein regulated during the critical period for song learning in the zebra finch. Neuron.

[27] Davidson W.S., Jonas A., Clayton D.F., George J.M. (1998). Stabilization of a-Synuclein Secondary Structure upon Binding to Synthetic Membranes. J. Biol. Chem..

[28] Rodriguez J.A., Ivanova M.I., Sawaya M.R., Cascio D., Reyes F.E., Shi D., Sangwan S., Guenther E.L., Johnson L.M., Zhang M. (2015). Structure of the toxic core of α-synuclein from invisible crystals. Nature.

[29] Salveson P.J., Spencer R.K., Nowick J.S. (2016). X-ray crystallographic structure of oligomers formed by a toxic β-hairpin derived from α-synuclein: Trimers and higher-order oligomers. J. Am. Chem. Soc..

[30] Giasson B.I., Murray I.V., Trojanowski J.Q., Lee V.M.-Y. (2001). A hydrophobic stretch of 12 amino acid residues in the middle of α-synuclein is essential for filament assembly. J. Biol. Chem..

[31] Anderson E.N., Hirpa D., Zheng K.H., Banerjee R., Gunawardena S. (2020). The non-amyloidal component region of α-synuclein is important for α-synuclein transport within axons. Front. Cell. Neurosci..

[32] Park S.M., Jung H.Y., Chung K.C., Rhim H., Park J.H., Kim J. (2002). Stress-Induced Aggregation Profiles of GST− α-Synuclein Fusion Proteins: Role of the C-Terminal Acidic Tail of α-Synuclein in Protein Thermosolubility and Stability. Biochemistry.

[33] Kim T.D., Paik S.R., Yang C.-H. (2002). Structural and functional implications of C-terminal regions of α-synuclein. Biochemistry.

[34] Farzadfard A., Pedersen J.N., Meisl G., Somavarapu A.K., Alam P., Goksøyr L., Nielsen M.A., Sander A.F., Knowles T.P., Pedersen J.S. (2022). The C-terminal tail of α-synuclein protects against aggregate replication but is critical for oligomerization. Commun. Biol..

[35] Flagmeier P., Meisl G., Vendruscolo M., Knowles T.P., Dobson C.M., Buell A.K., Galvagnion C. (2016). Mutations associated with familial Parkinson’s disease alter the initiation and amplification steps of α-synuclein aggregation. Proc. Natl. Acad. Sci. USA.

[36] Zunke F., Moise A.C., Belur N.R., Gelyana E., Stojkovska I., Dzaferbegovic H., Toker N.J., Jeon S., Fredriksen K., Mazzulli J.R. (2018). Reversible Conformational Conversion of alpha-Synuclein into Toxic Assemblies by Glucosylceramide. Neuron.

[37] Tracey T., Kirk S., Steyn F., Ngo S. (2021). The role of lipids in the central nervous system and their pathological implications in amyotrophic lateral sclerosis. Semin. Cell Dev. Biol..

[38] Vandenheuvel F.A. (1963). Study of biological structure at the molecular level with stereomodel projections I. The lipids in the myelin sheath of nerve. J. Am. Oil Chem. Soc..

[39] Eliezer D., Kutluay E., Bussell R., Browne G. (2001). Conformational properties of α-synuclein in its free and lipid-associated states. J. Mol. Biol..

[40] Bussell Jr R., Eliezer D. (2003). A structural and functional role for 11-mer repeats in α-synuclein and other exchangeable lipid binding proteins. J. Mol. Biol..

[41] Willingham S., Outeiro T.F., DeVit M.J., Lindquist S.L., Muchowski P.J. (2003). Yeast genes that enhance the toxicity of a mutant huntingtin fragment or α-synuclein. Science.

[42] Nuber S., Nam A.Y., Rajsombath M.M., Cirka H., Hronowski X., Wang J., Hodgetts K., Kalinichenko L.S., Müller C.P., Lambrecht V. (2021). A Stearoyl–Coenzyme A Desaturase Inhibitor Prevents Multiple Parkinson Disease Phenotypes in α-Synuclein Mice. Ann. Neurol..

[43] Pranav K., Usha M. (2014). Life Sciences Fundamental and Practice Part-1.

[44] Morell P., Toews A.D. (1996). Biochemistry of lipids. Handb. Clin. Neurol..

[45] Williams K.A., Deber C.M. (1993). The structure and function of central nervous system myelin. Crit. Rev. Clin. Lab. Sci..

[46] Masoro E.J. (1977). Lipids and Lipid Metabolism. Ann. Rev. Physioi..

[47] Fahy E., Cotter D., Sud M., Subramaniam S. (2011). Lipid classification, structures and tools. Biochim. Biophys. Acta (BBA)-Mol. Cell Biol. Lipids.

[48] Rustan A.C., Drevon C.A. (2001). Fatty acids: Structures and properties. e LS.

[49] Spector R. (1988). Fatty acid transport through the blood-brain barrier. J. Neurochem..

[50] Abbott N.J., Patabendige A.A., Dolman D.E., Yusof S.R., Begley D.J. (2010). Structure and function of the blood-brain barrier. Neurobiol. Dis..

[51] Jurevics H., Morell P. (1995). Cholesterol for synthesis of myelin is made locally, not imported into brain. J. Neurochem..

[52] Saher G., Stumpf S.K. (2015). Cholesterol in myelin biogenesis and hypomyelinating disorders. Biochim. Biophys. Acta.

[53] Hofmann K., Rodriguez-Rodriguez R., Gaebler A., Casals N., Scheller A., Kuerschner L. (2017). Astrocytes and oligodendrocytes in grey and white matter regions of the brain metabolize fatty acids. Sci. Rep..

[54] van Deijk A.L.F., Camargo N., Timmerman J., Heistek T., Brouwers J.F., Mogavero F., Mansvelder H.D., Smit A.B., Verheijen M.H. (2017). Astrocyte lipid metabolism is critical for synapse development and function in vivo. Glia.

[55] Camargo N., Goudriaan A., van Deijk A.-L.F., Otte W.M., Brouwers J.F., Lodder H., Gutmann D.H., Nave K.-A., Dijkhuizen R.M., Mansvelder H.D. (2017). Oligodendroglial myelination requires astrocyte-derived lipids. PLoS Biol..

[56] Chen J., Zhang X., Kusumo H., Costa L.G., Guizzetti M. (2013). Cholesterol efflux is differentially regulated in neurons and astrocytes: Implications for brain cholesterol homeostasis. Biochim. Biophys. Acta.

[57] Hirsch-Reinshagen V., Zhou S., Burgess B.L., Bernier L., McIsaac S.A., Chan J.Y., Tansley G.H., Cohn J.S., Hayden M.R., Wellington C.L. (2004). Deficiency of ABCA1 impairs apolipoprotein E metabolism in brain. J. Biol. Chem..

[58] Swanson L.W., Simmons D.M., Hofmann S.L., Goldstein J.L., Brown M.S. (1988). Localization of mRNA for low density lipoprotein receptor and a cholesterol synthetic enzyme in rabbit nervous system by in situ hybridization. Proc. Natl. Acad. Sci. USA.

[59] Pitas R., Boyles J., Lee S., Hui D., Weisgraber K. (1987). Lipoproteins and their receptors in the central nervous system. Characterization of the lipoproteins in cerebrospinal fluid and identification of apolipoprotein B, E (LDL) receptors in the brain. J. Biol. Chem..

[60] Zhao S., Hu X., Park J., Zhu Y., Zhu Q., Li H., Luo C., Han R., Cooper N., Qiu M. (2007). Selective expression of LDLR and VLDLR in myelinating oligodendrocytes. Dev. Dyn. Off. Publ. Am. Assoc. Anat..

[61] Simons M., Krämer E.-M., Thiele C., Stoffel W., Trotter J. (2000). Assembly of Myelin by Association of Proteolipid Protein with Cholesterol- and Galactosylceramid-rich Membrane Domains. J. Cell Biol..

[62] Werner H.B., Krämer-Albers E.M., Strenzke N., Saher G., Tenzer S., Ohno-Iwashita Y., De Monasterio-Schrader P., Möbius W., Moser T., Griffiths I.R. (2013). A critical role for the cholesterol-associated proteolipids PLP and M6B in myelination of the central nervous system. Glia.

[63] Garcia Corrales A.V., Haidar M., Bogie J.F.J., Hendriks J.J.A. (2021). Fatty Acid Synthesis in Glial Cells of the CNS. Int. J. Mol. Sci..

[64] Moore S.A. (2001). Polyunsaturated fatty acid synthesis and release by brain-derived cells in vitro. J. Mol. Neurosci..

[65] Bazan N. (1990). Supply of n-3 polyunsaturated fatty acids and their significance in the central nervous system. Nutr. Brain.

[66] Chandel N.S. (2021). Lipid metabolism. Cold Spring Harb. Perspect. Biol..

[67] Ralhan I., Chang C.-L., Lippincott-Schwartz J., Ioannou M.S. (2021). Lipid droplets in the nervous system. J. Cell Biol..

[68] Unger R.H., Clark G.O., Scherer P.E., Orci L. (2010). Lipid homeostasis, lipotoxicity and the metabolic syndrome. Biochim. Biophys. Acta (BBA)-Mol. Cell Biol. Lipids.

[69] Rambold A.S., Cohen S., Lippincott-Schwartz J. (2015). Fatty acid trafficking in starved cells: Regulation by lipid droplet lipolysis, autophagy, and mitochondrial fusion dynamics. Dev. Cell.

[70] Cabodevilla A., Sánchez-Caballero L., Picatoste F., Gubern A., Claro E. (2014). Cell survival during complete nutrient deprivation depends on lipid droplet-fueled β-oxidation of fatty acids (577.3). FASEB J..

[71] Edmond J., Robbins R., Bergstrom J., Cole R., De Vellis J. (1987). Capacity for substrate utilization in oxidative metabolism by neurons, astrocytes, and oligodendrocytes from developing brain in primary culture. J. Neurosci. Res..

[72] Hannun Y.A., Obeid L.M. (2018). Sphingolipids and their metabolism in physiology and disease. Nat. Rev. Mol. Cell Biol..

[73] Tracey T.J., Steyn F.J., Wolvetang E.J., Ngo S.T. (2018). Neuronal Lipid Metabolism: Multiple Pathways Driving Functional Outcomes in Health and Disease. Front. Mol. Neurosci..

[74] Burré J. (2015). The synaptic function of α-synuclein. J. Park. Dis..

[75] Spillantini M.G., Schmidt M.L., Lee V.M.-Y., Trojanowski J.Q., Jakes R., Goedert M. (1997). α-Synuclein in Lewy bodies. Nature.

[76] Jensen P.H., Nielsen M.S., Jakes R., Dotti C.G., Goedert M. (1998). Binding of α-synuclein to brain vesicles is abolished by familial Parkinson’s disease mutation. J. Biol. Chem..

[77] Segrest J.P., Jackson R.L., Morrisett J.D., Gotto Jr A.M. (1974). A molecular theory of lipid—Protein interactions in the plasma lipoproteins. FEBS Lett..

[78] van Rooijen B.D., Claessens M.M., Subramaniam V. (2009). Lipid bilayer disruption by oligomeric α-synuclein depends on bilayer charge and accessibility of the hydrophobic core. Biochim. Biophys. Acta (BBA)-Biomembr..

[79] Jo E., McLaurin J., Yip C.M., George-Hyslop P.S., Fraser P.E. (2000). α-Synuclein membrane interactions and lipid specificity. J. Biol. Chem..

[80] Rhoades E., Ramlall T.F., Webb W.W., Eliezer D. (2006). Quantification of α-synuclein binding to lipid vesicles using fluorescence correlation spectroscopy. Biophys. J..

[81] Narayanan V., Guo Y., Scarlata S. (2005). Fluorescence studies suggest a role for α-synuclein in the phosphatidylinositol lipid signaling pathway. Biochemistry.

[82] Fantini J., Carlus D., Yahi N. (2011). The fusogenic tilted peptide (67-78) of alpha-synuclein is a cholesterol binding domain. Biochim. Biophys. Acta.

[83] Fantini J., Yahi N. (2011). Molecular basis for the glycosphingolipid-binding specificity of alpha-synuclein: Key role of tyrosine 39 in membrane insertion. J. Mol. Biol..

[84] Wang G.F., Li C., Pielak G.J. (2010). 19F NMR studies of alpha-synuclein-membrane interactions. Protein Sci..

[85] Fortin D.L., Troyer M.D., Nakamura K., Kubo S., Anthony M.D., Edwards R.H. (2004). Lipid rafts mediate the synaptic localization of alpha-synuclein. J. Neurosci..

[86] Li W.-W., Yang R., Guo J.-C., Ren H.-M., Zha X.-L., Cheng J.-S., Cai D.-F. (2007). Localization of α-synuclein to mitochondria within midbrain of mice. Neuroreport.

[87] Bodner C.R., Maltsev A.S., Dobson C.M., Bax A. (2010). Differential phospholipid binding of α-synuclein variants implicated in Parkinson’s disease revealed by solution NMR spectroscopy. Biochemistry.

[88] Fusco G., De Simone A., Gopinath T., Vostrikov V., Vendruscolo M., Dobson C.M., Veglia G. (2014). Direct observation of the three regions in α-synuclein that determine its membrane-bound behaviour. Nat. Commun..

[89] Bartels T., Ahlstrom L.S., Leftin A., Kamp F., Haass C., Brown M.F., Beyer K. (2010). The N-terminus of the intrinsically disordered protein α-synuclein triggers membrane binding and helix folding. Biophys. J..

[90] Burré J., Sharma M., Tsetsenis T., Buchman V., Etherton M.R., Südhof T.C. (2010). α-Synuclein promotes SNARE-complex assembly in vivo and in vitro. Science.

[91] Fusco G., Pape T., Stephens A.D., Mahou P., Costa A.R., Kaminski C.F., Kaminski Schierle G.S., Vendruscolo M., Veglia G., Dobson C.M. (2016). Structural basis of synaptic vesicle assembly promoted by α-synuclein. Nat. Commun..

[92] Cremades N., Cohen S.I., Deas E., Abramov A.Y., Chen A.Y., Orte A., Sandal M., Clarke R.W., Dunne P., Aprile F.A. (2012). Direct observation of the interconversion of normal and toxic forms of α-synuclein. Cell.

[93] Grey M., Linse S., Nilsson H., Brundin P., Sparr E. (2011). Membrane interaction of α-synuclein in different aggregation states. J. Park. Dis..

[94] Fanning S., Selkoe D., Dettmer U. (2020). Parkinson’s disease: Proteinopathy or lipidopathy?. NPJ Park. Dis..

[95] Klemann C., Martens G.J.M., Sharma M., Martens M.B., Isacson O., Gasser T., Visser J.E., Poelmans G. (2017). Integrated molecular landscape of Parkinson’s disease. NPJ Park. Dis..

[96] Golovko M.Y., Faergeman N.J., Cole N.B., Castagnet P.I., Nussbaum R.L., Murphy E.J. (2005). α-synuclein gene deletion decreases brain palmitate uptake and alters the palmitate metabolism in the absence of α-synuclein palmitate binding. Biochemistry.

[97] Golovko M.Y., Rosenberger T.A., Faergeman N.J., Feddersen S., Cole N.B., Pribill I., Berger J., Nussbaum R.L., Murphy E.J. (2006). Acyl-CoA synthetase activity links wild-type but not mutant α-synuclein to brain arachidonate metabolism. Biochemistry.

[98] Golovko M.Y., Rosenberger T.A., Feddersen S., Færgeman N.J., Murphy E.J. (2007). α-Synuclein gene ablation increases docosahexaenoic acid incorporation and turnover in brain phospholipids. J. Neurochem..

[99] Campos S.S., Alza N.P., Salvador G.A. (2018). Lipid metabolism alterations in the neuronal response to A53T α-synuclein and Fe-induced injury. Arch. Biochem. Biophys..

[100] Barceló-Coblijn G., Golovko M.Y., Weinhofer I., Berger J., Murphy E.J. (2007). Brain neutral lipids mass is increased in α-synuclein gene-ablated mice. J. Neurochem..

[101] Ruipérez V., Darios F., Davletov B. (2010). Alpha-synuclein, lipids and Parkinson’s disease. Prog. Lipid Res..

[102] Xicoy H., Wieringa B., Martens G.J. (2019). The role of lipids in Parkinson’s disease. Cells.

[103] Jin U., Park S.J., Park S.M. (2019). Cholesterol metabolism in the brain and its association with Parkinson’s disease. Exp. Neurobiol..

[104] Huang X., Sterling N.W., Du G., Sun D., Stetter C., Kong L., Zhu Y., Neighbors J., Lewis M.M., Chen H. (2019). Brain cholesterol metabolism and Parkinson’s disease. Mov. Disord..

[105] Indellicato R., Trinchera M. (2019). The link between Gaucher disease and Parkinson’s disease sheds light on old and novel disorders of sphingolipid metabolism. Int. J. Mol. Sci..

[106] Quinville B.M., Deschenes N.M., Ryckman A.E., Walia J.S. (2021). A comprehensive review: Sphingolipid metabolism and implications of disruption in sphingolipid homeostasis. Int. J. Mol. Sci..

[107] Fanning S., Haque A., Imberdis T., Baru V., Barrasa M.I., Nuber S., Termine D., Ramalingam N., Ho G.P.H., Noble T. (2019). Lipidomic Analysis of alpha-Synuclein Neurotoxicity Identifies Stearoyl CoA Desaturase as a Target for Parkinson Treatment. Mol. Cell.

[108] Yoo D., Lim Y., Son Y., Rho H., Shin C., Ahn T.-B. (2021). Dietary intake and plasma levels of polyunsaturated fatty acids in early-stage Parkinson’s disease. Sci. Rep..

[109] Assayag K., Yakunin E., Loeb V., Selkoe D.J., Sharon R. (2007). Polyunsaturated fatty acids induce α-synuclein-related pathogenic changes in neuronal cells. Am. J. Pathol..

[110] Sharon R., Bar-Joseph I., Mirick G.E., Serhan C.N., Selkoe D.J. (2003). Altered fatty acid composition of dopaminergic neurons expressing α-synuclein and human brains with α-synucleinopathies. J. Biol. Chem..

[111] Cheng D., Jenner A.M., Shui G., Cheong W.F., Mitchell T.W., Nealon J.R., Kim W.S., McCann H., Wenk M.R., Halliday G.M. (2011). Lipid pathway alterations in Parkinson’s disease primary visual cortex. PLoS ONE.

[112] García-Sanz P., Orgaz L., Fuentes J.M., Vicario C., Moratalla R. (2018). Cholesterol and multilamellar bodies: Lysosomal dysfunction in GBA-Parkinson disease. Autophagy.

[113] Paul R., Choudhury A., Kumar S., Giri A., Sandhir R., Borah A. (2017). Cholesterol contributes to dopamine-neuronal loss in MPTP mouse model of Parkinson’s disease: Involvement of mitochondrial dysfunctions and oxidative stress. PLoS ONE.

[114] Raju A., Jaisankar P., Borah A., Mohanakumar K.P. (2017). 1-methyl-4-phenylpyridinium-induced death of differentiated SH-SY5Y neurons is potentiated by cholesterol. Ann. Neurosci..

[115] Mullin S., Hughes D., Mehta A., Schapira A. (2019). Neurological effects of glucocerebrosidase gene mutations. Eur. J. Neurol..

[116] McCampbell A., Truong D., Broom D.C., Allchorne A., Gable K., Cutler R.G., Mattson M.P., Woolf C.J., Frosch M.P., Harmon J.M. (2005). Mutant SPTLC1 dominantly inhibits serine palmitoyltransferase activity in vivo and confers an age-dependent neuropathy. Hum. Mol. Genet.

[117] Simons K., Ikonen E. (1997). Functional rafts in cell membranes. Nature.

[118] Hannun Y.A., Obeid L.M. (2008). Principles of bioactive lipid signalling: Lessons from sphingolipids. Nat. Rev. Mol. Cell Biol..

[119] Futerman A.H., Riezman H. (2005). The ins and outs of sphingolipid synthesis. Trends Cell Biol..

[120] Farmer K., Smith C.A., Hayley S., Smith J. (2015). Major alterations of phosphatidylcholine and lysophosphotidylcholine lipids in the substantia nigra using an early stage model of Parkinson’s disease. Int. J. Mol. Sci..

[121] Lobasso S., Tanzarella P., Vergara D., Maffia M., Cocco T., Corcelli A. (2017). Lipid profiling of parkin-mutant human skin fibroblasts. J. Cell. Physiol..

[122] Cunningham T.J., Yao L., Lucena A. (2008). Product inhibition of secreted phospholipase A2 may explain lysophosphatidylcholines’ unexpected therapeutic properties. J. Inflamm..

[123] Riekkinen P., Rinne U.K., Pelliniemi T.-T., Sonninen V. (1975). Interaction between dopamine and phospholipids: Studies of the substantia nigra in parkinson disease patients. Arch. Neurol..

[124] Hattingen E., Magerkurth J., Pilatus U., Mozer A., Seifried C., Steinmetz H., Zanella F., Hilker R. (2009). Phosphorus and proton magnetic resonance spectroscopy demonstrates mitochondrial dysfunction in early and advanced Parkinson’s disease. Brain.

[125] Wang S., Zhang S., Liou L.-C., Ren Q., Zhang Z., Caldwell G.A., Caldwell K.A., Witt S.N. (2014). Phosphatidylethanolamine deficiency disrupts α-synuclein homeostasis in yeast and worm models of Parkinson disease. Proc. Natl. Acad. Sci. USA.

[126] Wood P.L., Tippireddy S., Feriante J., Woltjer R.L. (2018). Augmented frontal cortex diacylglycerol levels in Parkinson’s disease and Lewy Body Disease. PLoS ONE.

[127] Nakamura K., Nemani V.M., Wallender E.K., Kaehlcke K., Ott M., Edwards R.H. (2008). Optical reporters for the conformation of α-synuclein reveal a specific interaction with mitochondria. J. Neurosci..

[128] Parihar M., Parihar A., Fujita M., Hashimoto M., Ghafourifar P. (2008). Mitochondrial association of alpha-synuclein causes oxidative stress. Cell. Mol. Life Sci..

[129] Banerjee K., Sinha M., Pham C.L.L., Jana S., Chanda D., Cappai R., Chakrabarti S. (2010). α-Synuclein induced membrane depolarization and loss of phosphorylation capacity of isolated rat brain mitochondria: Implications in Parkinson’s disease. FEBS Lett..

[130] Bodner C.R., Dobson C.M., Bax A. (2009). Multiple tight phospholipid-binding modes of α-synuclein revealed by solution NMR spectroscopy. J. Mol. Biol..

[131] Pancoe S.X., Wang Y.J., Shimogawa M., Perez R.M., Giannakoulias S., Petersson E.J. (2022). Effects of Mutations and Post-Translational Modifications on α-Synuclein In Vitro Aggregation. J. Mol. Biol..

[132] Daida K., Shimonaka S., Shiba-Fukushima K., Ogata J., Yoshino H., Okuzumi A., Hatano T., Motoi Y., Hirunagi T., Katsuno M. (2022). α-Synuclein V15A Variant in Familial Parkinson’s Disease Exhibits a Weaker Lipid-Binding Property. Mov. Disord..

[133] Grassel A., Borland C., Bertolotti F., Osselborn R., Nassuna T., Zabat B., DebBurman S. (2022). Insight into Parkinson’s Disease From a Yeast Model: How Three Alpha-Synuclein Mutants (A18T, A29S, & A53V) Generate Toxicity. FASEB J..

[134] Kim Y.S., Laurine E., Woods W., Lee S.-J. (2006). A novel mechanism of interaction between α-synuclein and biological membranes. J. Mol. Biol..

[135] Kuwahara T., Tonegawa R., Ito G., Mitani S., Iwatsubo T. (2012). Phosphorylation of α-synuclein protein at Ser-129 reduces neuronal dysfunction by lowering its membrane binding property in Caenorhabditis elegans. J. Biol. Chem..

[136] Ruf V.C., Nubling G.S., Willikens S., Shi S., Schmidt F., Levin J., Botzel K., Kamp F., Giese A. (2019). Different Effects of alpha-Synuclein Mutants on Lipid Binding and Aggregation Detected by Single Molecule Fluorescence Spectroscopy and ThT Fluorescence-Based Measurements. ACS Chem. Neurosci..

[137] Rovere M., Powers A.E., Jiang H., Pitino J.C., Fonseca-Ornelas L., Patel D.S., Achille A., Langen R., Varkey J., Bartels T. (2019). E46K-like alpha-synuclein mutants increase lipid interactions and disrupt membrane selectivity. J. Biol. Chem..

[138] Fredenburg R.A., Rospigliosi C., Meray R.K., Kessler J.C., Lashuel H.A., Eliezer D., Lansbury P.T. (2007). The impact of the E46K mutation on the properties of α-synuclein in its monomeric and oligomeric states. Biochemistry.

[139] Khalaf O., Fauvet B., Oueslati A., Dikiy I., Mahul-Mellier A.L., Ruggeri F.S., Mbefo M.K., Vercruysse F., Dietler G., Lee S.J. (2014). The H50Q mutation enhances alpha-synuclein aggregation, secretion, and toxicity. J. Biol. Chem..

[140] Mohite G.M., Kumar R., Panigrahi R., Navalkar A., Singh N., Datta D., Mehra S., Ray S., Gadhe L.G., Das S. (2018). Comparison of kinetics, toxicity, oligomer formation, and membrane binding capacity of α-synuclein familial mutations at the A53 site, including the newly discovered A53V mutation. Biochemistry.

[141] Ghosh D., Sahay S., Ranjan P., Salot S., Mohite G.M., Singh P.K., Dwivedi S., Carvalho E., Banerjee R., Kumar A. (2014). The newly discovered Parkinson’s disease associated Finnish mutation (A53E) attenuates α-synuclein aggregation and membrane binding. Biochemistry.

[142] Perissinotto F., Stani C., De Cecco E., Vaccari L., Rondelli V., Posocco P., Parisse P., Scaini D., Legname G., Casalis L. (2020). Iron-mediated interaction of alpha synuclein with lipid raft model membranes. Nanoscale.

[143] Xiang W., Menges S., Schlachetzki J., Meixner H., Hoffmann A.-C., Schlötzer-Schrehardt U., Becker C.-M., Winkler J., Klucken J. (2015). Posttranslational modification and mutation of histidine 50 trigger alpha synuclein aggregation and toxicity. Mol. Neurodegener..

[144] Vilariño-Güell C., Wider C., Ross O.A., Dachsel J.C., Kachergus J.M., Lincoln S.J., Soto-Ortolaza A.I., Cobb S.A., Wilhoite G.J., Bacon J.A. (2011). VPS35 mutations in Parkinson disease. Am. J. Hum. Genet.

[145] Patel D., Witt S.N. (2018). Sorting Out the Role of α-Synuclein in Retromer-Mediated Endosomal Protein Sorting. J. Exp. Neurosci..

[146] Rajasekaran S., Peterson P.P., Liu Z., Robinson L.C., Witt S.N. (2022). α-synuclein inhibits Snx3-retromer retrograde trafficking of the conserved membrane-bound proprotein convertase Kex2 in the secretory pathway of Saccharomyces cerevisiae. Hum. Mol. Genet.

[147] He S., Wang F., Yung K.K.L., Zhang S., Qu S. (2021). Effects of α-Synuclein-associated post-translational modifications in Parkinson’s disease. ACS Chem. Neurosci..

[148] Schaffert L.-N., Carter W.G. (2020). Do post-translational modifications influence protein aggregation in neurodegenerative diseases: A systematic review. Brain Sci..

[149] Delenclos M., Burgess J.D., Lamprokostopoulou A., Outeiro T.F., Vekrellis K., McLean P.J. (2019). Cellular models of alpha-synuclein toxicity and aggregation. J. Neurochem..

[150] Gadhavi J., Patel M., Bhatia D., Gupta S. (2022). Neurotoxic or neuroprotective: Post-translational modifications of α-synuclein at the cross-roads of functions. Biochimie.

[151] Oueslati A., Fournier M., Lashuel H.A. (2010). Role of post-translational modifications in modulating the structure, function and toxicity of α-synuclein: Implications for Parkinson’s disease pathogenesis and therapies. Prog. Brain Res..

[152] Mahul-Mellier A.-L., Fauvet B., Gysbers A., Dikiy I., Oueslati A., Georgeon S., Lamontanara A.J., Bisquertt A., Eliezer D., Masliah E. (2014). c-Abl phosphorylates α-synuclein and regulates its degradation: Implication for α-synuclein clearance and contribution to the pathogenesis of Parkinson’s disease. Hum. Mol. Genet.

[153] Paleologou K.E., Oueslati A., Shakked G., Rospigliosi C.C., Kim H.-Y., Lamberto G.R., Fernandez C.O., Schmid A., Chegini F., Gai W.P. (2010). Phosphorylation at S87 is enhanced in synucleinopathies, inhibits α-synuclein oligomerization, and influences synuclein-membrane interactions. J. Neurosci..

[154] Nubling G.S., Levin J., Bader B., Lorenzl S., Hillmer A., Hogen T., Kamp F., Giese A. (2014). Modelling Ser129 phosphorylation inhibits membrane binding of pore-forming alpha-synuclein oligomers. PLoS ONE.

[155] Ma M.-R., Hu Z.-W., Zhao Y.-F., Chen Y.-X., Li Y.-M. (2016). Phosphorylation induces distinct alpha-synuclein strain formation. Sci. Rep..

[156] Samuel F., Flavin W.P., Iqbal S., Pacelli C., Renganathan S.D.S., Trudeau L.-E., Campbell E.M., Fraser P.E., Tandon A. (2016). Effects of serine 129 phosphorylation on α-synuclein aggregation, membrane association, and internalization. J. Biol. Chem..

[157] Runfola M., De Simone A., Vendruscolo M., Dobson C.M., Fusco G. (2020). The N-terminal acetylation of α-synuclein changes the affinity for lipid membranes but not the structural properties of the bound state. Sci. Rep..

[158] Sevcsik E., Trexler A.J., Dunn J.M., Rhoades E. (2011). Allostery in a disordered protein: Oxidative modifications to α-synuclein act distally to regulate membrane binding. J. Am. Chem. Soc..

[159] Lewis Y.E., Abeywardana T., Lin Y.H., Galesic A., Pratt M.R. (2016). Synthesis of a Bis-thio-acetone (BTA) Analogue of the Lysine Isopeptide Bond and its Application to Investigate the Effects of Ubiquitination and SUMOylation on α-Synuclein Aggregation and Toxicity. ACS Chem. Biol..

[160] Hejjaoui M., Haj-Yahya M., Kumar K.A., Brik A., Lashuel H.A. (2011). Towards Elucidation of the Role of Ubiquitination in the Pathogenesis of Parkinson’s Disease with Semisynthetic Ubiquitinated α-Synuclein. Angew. Chem. Int. Ed..

[161] Caparotta M., Bustos D.M., Masone D. (2020). Order–disorder skewness in alpha-synuclein: A key mechanism to recognize membrane curvature. Phys. Chem. Chem. Phys..

[162] van der Wateren I.M., Knowles T.P., Buell A.K., Dobson C.M., Galvagnion C. (2018). C-terminal truncation of α-synuclein promotes amyloid fibril amplification at physiological pH. Chem. Sci..

[163] Flynn J.D., Gimmen M.Y., Dean D.N., Lacy S.M., Lee J.C. (2020). Terminal Alkynes as Raman Probes of α-Synuclein in Solution and in Cells. ChemBioChem.

[164] Faustini G., Longhena F., Bruno A., Bono F., Grigoletto J., La Via L., Barbon A., Casiraghi A., Straniero V., Valoti E. (2020). Alpha-synuclein/synapsin III pathological interplay boosts the motor response to methylphenidate. Neurobiol. Dis..

[165] Ma L., Yang C., Zhang X., Li Y., Wang S., Zheng L., Huang K. (2018). C-terminal truncation exacerbates the aggregation and cytotoxicity of α-Synuclein: A vicious cycle in Parkinson’s disease. Biochim. Biophys. Acta (BBA)-Mol. Basis Dis..

[166] Levine P.M., Galesic A., Balana A.T., Mahul-Mellier A.-L., Navarro M.X., De Leon C.A., Lashuel H.A., Pratt M.R. (2019). α-Synuclein O-GlcNAcylation alters aggregation and toxicity, revealing certain residues as potential inhibitors of Parkinson’s disease. Proc. Natl. Acad. Sci. USA.

[167] Uceda A.B., Frau J., Vilanova B., Adrover M. (2022). Glycation of alpha-synuclein hampers its binding to synaptic-like vesicles and its driving effect on their fusion. Cell Mol. Life Sci..

[168] Manning G., Whyte D.B., Martinez R., Hunter T., Sudarsanam S. (2002). The protein kinase complement of the human genome. Science.

[169] Cohen P. (1989). The structure and regulation of protein phosphatases. Annu. Rev. Biochem..

[170] Cohen P. (2002). The origins of protein phosphorylation. Nat. Cell Biol..

[171] Anderson J.P., Walker D.E., Goldstein J.M., De Laat R., Banducci K., Caccavello R.J., Barbour R., Huang J., Kling K., Lee M. (2006). Phosphorylation of Ser-129 is the dominant pathological modification of α-synuclein in familial and sporadic Lewy body disease. J. Biol. Chem..

[172] Kosten J., Binolfi A., Stuiver M., Verzini S., Theillet F.-X., Bekei B., van Rossum M., Selenko P. (2014). Efficient modification of alpha-synuclein serine 129 by protein kinase CK1 requires phosphorylation of tyrosine 125 as a priming event. ACS Chem. Neurosci..

[173] Zheng W., Zhang Z., Ye Y., Wu Q., Liu M., Li C. (2019). Phosphorylation dependent α-synuclein degradation monitored by in-cell NMR. Chem. Commun..

[174] Schreurs S., Gerard M., Derua R., Waelkens E., Taymans J.-M., Baekelandt V., Engelborghs Y. (2014). In vitro phosphorylation does not influence the aggregation kinetics of WT α-synuclein in contrast to its phosphorylation mutants. Int. J. Mol. Sci..

[175] Brahmachari S., Ge P., Lee S.H., Kim D., Karuppagounder S.S., Kumar M., Mao X., Shin J.H., Lee Y., Pletnikova O. (2016). Activation of tyrosine kinase c-Abl contributes to α-synuclein–induced neurodegeneration. J. Clin. Investig..

[176] McFarland N.R., Fan Z., Xu K., Schwarzschild M.A., Feany M.B., Hyman B.T., McLean P.J. (2009). α-Synuclein S129 phosphorylation mutants do not alter nigrostriatal toxicity in a rat model of parkinson disease. J. Neuropathol. Exp. Neurol..

[177] Dikiy I., Eliezer D. (2014). N-terminal acetylation stabilizes N-terminal helicity in lipid- and micelle-bound alpha-synuclein and increases its affinity for physiological membranes. J. Biol. Chem..

[178] Bhattacharjee P., Öhrfelt A., Lashley T., Blennow K., Brinkmalm A., Zetterberg H. (2019). Mass spectrometric analysis of Lewy body-enriched α-synuclein in Parkinson’s disease. J. Proteome Res..

[179] Kang L., Moriarty G.M., Woods L.A., Ashcroft A.E., Radford S.E., Baum J. (2012). N-Terminal acetylation of α-synuclein induces increased transient helical propensity and decreased aggregation rates in the intrinsically disordered monomer. Protein Sci..

[180] O’Leary E.I., Jiang Z., Strub M.-P., Lee J.C. (2018). Effects of phosphatidylcholine membrane fluidity on the conformation and aggregation of N-terminally acetylated α-synuclein. J. Biol. Chem..

[181] Good P.F., Hsu A., Werner P., Perl D.P., Olanow C.W. (1998). Protein nitration in Parkinson’s disease. J. Neuropathol. Exp. Neurol..

[182] Paxinou E., Chen Q., Weisse M., Giasson B.I., Norris E.H., Rueter S.M., Trojanowski J.Q., Lee V.M.-Y., Ischiropoulos H. (2001). Induction of α-synuclein aggregation by intracellular nitrative insult. J. Neurosci..

[183] Giasson B.I., Duda J.E., Murray I.V., Chen Q., Souza J.M., Hurtig H.I., Ischiropoulos H., Trojanowski J.Q., Lee V.M. (2000). Oxidative damage linked to neurodegeneration by selective α-synuclein nitration in synucleinopathy lesions. Science.

[184] Xiang W., Schlachetzki J.C., Helling S., Bussmann J.C., Berlinghof M., Schäffer T.E., Marcus K., Winkler J., Klucken J., Becker C.-M. (2013). Oxidative stress-induced posttranslational modifications of alpha-synuclein: Specific modification of alpha-synuclein by 4-hydroxy-2-nonenal increases dopaminergic toxicity. Mol. Cell. Neurosci..

[185] Liu Y., Qiang M., Wei Y., He R. (2011). A novel molecular mechanism for nitrated α-synuclein-induced cell death. J. Mol. Cell. Biol..

[186] Yu Z., Xu X., Xiang Z., Zhou J., Zhang Z., Hu C., He C. (2010). Nitrated α-synuclein induces the loss of dopaminergic neurons in the substantia nigra of rats. PLoS ONE.

[187] Rape M., Komander D. (2012). The ubiquitin code. Annu. Rev. Biochem..

[188] Kuzuhara S., Mori H., Izumiyama N., Yoshimura M., Ihara Y. (1988). Lewy bodies are ubiquitinated. Acta Neuropathol..

[189] Meier F., Abeywardana T., Dhall A., Marotta N.P., Varkey J., Langen R., Chatterjee C., Pratt M.R. (2012). Semisynthetic, site-specific ubiquitin modification of α-synuclein reveals differential effects on aggregation. J. Am. Chem. Soc..

[190] Iyer A., Claessens M.M. (2019). Disruptive membrane interactions of alpha-synuclein aggregates. Biochim. Biophys. Acta (BBA)-Proteins Proteom..

[191] Kim K.S., Choi Y.R., Park J.-Y., Lee J.-H., Kim D.K., Lee S.-J., Paik S.R., Jou I., Park S.M. (2012). Proteolytic cleavage of extracellular α-synuclein by plasmin: Implications for Parkinson disease. J. Biol. Chem..

[192] Iwata A., Maruyama M., Akagi T., Hashikawa T., Kanazawa I., Tsuji S., Nukina N. (2003). Alpha-synuclein degradation by serine protease neurosin: Implication for pathogenesis of synucleinopathies. Hum. Mol. Genet.

[193] Sevlever D., Jiang P., Yen S.-H.C. (2008). Cathepsin D is the main lysosomal enzyme involved in the degradation of α-synuclein and generation of its carboxy-terminally truncated species. Biochemistry.

[194] Wang W., Nguyen L.T., Burlak C., Chegini F., Guo F., Chataway T., Ju S., Fisher O.S., Miller D.W., Datta D. (2016). Caspase-1 causes truncation and aggregation of the Parkinson’s disease-associated protein α-synuclein. Proc. Natl. Acad. Sci. USA.

[195] Diepenbroek M., Casadei N., Esmer H., Saido T.C., Takano J., Kahle P.J., Nixon R.A., Rao M.V., Melki R., Pieri L. (2014). Overexpression of the calpain-specific inhibitor calpastatin reduces human alpha-Synuclein processing, aggregation and synaptic impairment in [A30P] αSyn transgenic mice. Hum. Mol. Genet.

[196] Terada M., Suzuki G., Nonaka T., Kametani F., Tamaoka A., Hasegawa M. (2018). The effect of truncation on prion-like properties of α-synuclein. J. Biol. Chem..

[197] Kellie J.F., Higgs R.E., Ryder J.W., Major A., Beach T.G., Adler C.H., Merchant K., Knierman M.D. (2014). Quantitative measurement of intact alpha-synuclein proteoforms from post-mortem control and Parkinson’s disease brain tissue by intact protein mass spectrometry. Sci. Rep..

[198] Sorrentino Z.A., Giasson B.I. (2020). The emerging role of α-synuclein truncation in aggregation and disease. J. Biol. Chem..

[199] Öhrfelt A., Zetterberg H., Andersson K., Persson R., Secic D., Brinkmalm G., Wallin A., Mulugeta E., Francis P.T., Vanmechelen E. (2011). Identification of novel α-synuclein isoforms in human brain tissue by using an online nanoLC-ESI-FTICR-MS method. Neurochem. Res..

[200] Murray I.V., Giasson B.I., Quinn S.M., Koppaka V., Axelsen P.H., Ischiropoulos H., Trojanowski J.Q., Lee V.M.-Y. (2003). Role of α-synuclein carboxy-terminus on fibril formation in vitro. Biochemistry.

[201] Michell A.W., Tofaris G., Gossage H., Tyers P., Spillantini M., Barker R. (2007). The effect of truncated human α-synuclein (1–120) on dopaminergic cells in a transgenic mouse model of Parkinson’s disease. Cell Transplant..

[202] Sorrentino Z.A., Vijayaraghavan N., Gorion K.-M., Riffe C.J., Strang K.H., Caldwell J., Giasson B.I. (2018). Physiological C-terminal truncation of α-synuclein potentiates the prion-like formation of pathological inclusions. J. Biol. Chem..

[203] Torres C.-R., Hart G.W. (1984). Topography and polypeptide distribution of terminal N-acetylglucosamine residues on the surfaces of intact lymphocytes. Evidence for O-linked GlcNAc. J. Biol. Chem..

[204] Wang Z., Park K., Comer F., Hsieh-Wilson L.C., Saudek C.D., Hart G.W. (2009). Site-specific GlcNAcylation of human erythrocyte proteins: Potential biomarker (s) for diabetes. Diabetes.

[205] Alfaro J.F., Gong C.-X., Monroe M.E., Aldrich J.T., Clauss T.R., Purvine S.O., Wang Z., Camp D.G., Shabanowitz J., Stanley P. (2012). Tandem mass spectrometry identifies many mouse brain O-GlcNAcylated proteins including EGF domain-specific O-GlcNAc transferase targets. Proc. Natl. Acad. Sci. USA.

[206] Wang S., Yang F., Petyuk V.A., Shukla A.K., Monroe M.E., Gritsenko M.A., Rodland K.D., Smith R.D., Qian W.J., Gong C.X. (2017). Quantitative proteomics identifies altered O-GlcNAcylation of structural, synaptic and memory-associated proteins in Alzheimer’s disease. J. Pathol..

[207] Marotta N.P., Lin Y.H., Lewis Y.E., Ambroso M.R., Zaro B.W., Roth M.T., Arnold D.B., Langen R., Pratt M.R. (2015). O-GlcNAc modification blocks the aggregation and toxicity of the protein α-synuclein associated with Parkinson’s disease. Nat. Chem..

[208] Ryan P., Xu M., Davey A.K., Danon J.J., Mellick G.D., Kassiou M., Rudrawar S. (2019). O-GlcNAc modification protects against protein misfolding and aggregation in neurodegenerative disease. ACS Chem. Neurosci..

[209] Salahuddin P., Rabbani G., Khan R. (2014). The role of advanced glycation end products in various types of neurodegenerative disease: A therapeutic approach. Cell. Mol. Biol. Lett..

[210] Choi Y.-G., Lim S. (2010). Nε-(carboxymethyl) lysine linkage to α-synuclein and involvement of advanced glycation end products in α-synuclein deposits in an MPTP-intoxicated mouse model. Biochimie.

[211] Vicente Miranda H., Szegő É.M., Oliveira L.M., Breda C., Darendelioglu E., de Oliveira R.M., Ferreira D.G., Gomes M.A., Rott R., Oliveira M. (2017). Glycation potentiates α-synuclein-associated neurodegeneration in synucleinopathies. Brain.

[212] Bar-On P., Crews L., Koob A.O., Mizuno H., Adame A., Spencer B., Masliah E. (2008). Statins reduce neuronal α-synuclein aggregation in in vitro models of Parkinson’s disease. J. Neurochem..

[213] Dai L., Wang J., He M., Xiong M., Tian Y., Liu C., Zhang Z. (2021). Lovastatin Alleviates α-Synuclein Aggregation and Phosphorylation in Cellular Models of Synucleinopathy. Front. Mol. Neurosci..

[214] Koob A.O., Ubhi K., Paulsson J.F., Kelly J., Rockenstein E., Mante M., Adame A., Masliah E. (2010). Lovastatin ameliorates α-synuclein accumulation and oxidation in transgenic mouse models of α-synucleinopathies. Exp. Neurol..

[215] Selley M.L. (2005). Simvastatin prevents 1-methyl-4-phenyl-1, 2, 3, 6-tetrahydropyridine-induced striatal dopamine depletion and protein tyrosine nitration in mice. Brain Res..

[216] Ghosh A., Roy A., Matras J., Brahmachari S., Gendelman H.E., Pahan K. (2009). Simvastatin inhibits the activation of p21ras and prevents the loss of dopaminergic neurons in a mouse model of Parkinson’s disease. J. Neurosci..

[217] Mingione A., Pivari F., Plotegher N., Dei Cas M., Zulueta A., Bocci T., Trinchera M., Albi E., Maglione V., Caretti A. (2021). Inhibition of Ceramide Synthesis Reduces α-Synuclein Proteinopathy in a Cellular Model of Parkinson’s Disease. Int. J. Mol. Sci..

[218] Kumar S., Kumar R., Kumari M., Kumari R., Saha S., Bhavesh N.S., Maiti T.K. (2021). Ellagic Acid Inhibits alpha-Synuclein Aggregation at Multiple Stages and Reduces Its Cytotoxicity. ACS Chem. Neurosci..

[219] Perni M., Galvagnion C., Maltsev A., Meisl G., Müller M.B., Challa P.K., Kirkegaard J.B., Flagmeier P., Cohen S.I., Cascella R. (2017). A natural product inhibits the initiation of α-synuclein aggregation and suppresses its toxicity. Proc. Natl. Acad. Sci. USA.

[220] Hebron M.L., Lonskaya I., Moussa C.E.-H. (2013). Nilotinib reverses loss of dopamine neurons and improves motor behavior via autophagic degradation of α-synuclein in Parkinson’s disease models. Hum. Mol. Genet.

[221] Collins L.M., Adriaanse L.J., Theratile S.D., Hegarty S.V., Sullivan A.M., O’Keeffe G.W. (2015). Class-IIa histone deacetylase inhibition promotes the growth of neural processes and protects them against neurotoxic insult. Mol. Neurobiol..

[222] Bassil F., Fernagut P.-O., Bezard E., Pruvost A., Leste-Lasserre T., Hoang Q.Q., Ringe D., Petsko G.A., Meissner W.G. (2016). Reducing C-terminal truncation mitigates synucleinopathy and neurodegeneration in a transgenic model of multiple system atrophy. Proc. Natl. Acad. Sci. USA.

[223] Iljina M., Tosatto L., Choi M.L., Sang J.C., Ye Y., Hughes C.D., Bryant C.E., Gandhi S., Klenerman D. (2016). Arachidonic acid mediates the formation of abundant alpha-helical multimers of alpha-synuclein. Sci. Rep..

[224] Beal M.F. (2003). Bioenergetic approaches for neuroprotection in Parkinson’s disease. Ann. Neurol. Off. J. Am. Neurol. Assoc. Child. Neurol. Soc..

[225] Anderson D.W., Bradbury K.A., Schneider J.S. (2006). Neuroprotection in Parkinson models varies with toxin administration protocol. Eur. J. Neurosci..

[226] Jia H., Li X., Gao H., Feng Z., Li X., Zhao L., Jia X., Zhang H., Liu J. (2008). High doses of nicotinamide prevent oxidative mitochondrial dysfunction in a cellular model and improve motor deficit in a Drosophila model of Parkinson’s disease. J. Neurosci. Res..

[227] Devos D., Moreau C., Devedjian J.C., Kluza J., Petrault M., Laloux C., Jonneaux A., Ryckewaert G., Garçon G., Rouaix N. (2014). Targeting chelatable iron as a therapeutic modality in Parkinson’s disease. Antioxid. Redox Signal..

[228] Vincent B.M., Tardiff D.F., Piotrowski J.S., Aron R., Lucas M.C., Chung C.Y., Bacherman H., Chen Y., Pires M., Subramaniam R. (2018). Inhibiting stearoyl-CoA desaturase ameliorates α-synuclein cytotoxicity. Cell Rep..

[229] Liu X., Strable M.S., Ntambi J.M. (2011). Stearoyl CoA desaturase 1: Role in cellular inflammation and stress. Adv. Nutr..

[230] Imberdis T., Negri J., Ramalingam N., Terry-Kantor E., Ho G.P., Fanning S., Stirtz G., Kim T.-E., Levy O.A., Young-Pearse T.L. (2019). Cell models of lipid-rich α-synuclein aggregation validate known modifiers of α-synuclein biology and identify stearoyl-CoA desaturase. Proc. Natl. Acad. Sci. USA.

[231] Hubler Z., Allimuthu D., Bederman I., Elitt M.S., Madhavan M., Allan K.C., Shick H.E., Garrison E., Karl M.T., Factor D.C. (2018). Accumulation of 8, 9-unsaturated sterols drives oligodendrocyte formation and remyelination. Nature.

[232] Alberts A., Chen J., Kuron G., Hunt V., Huff J., Hoffman C., Rothrock J., Lopez M., Joshua H., Harris E. (1980). Mevinolin: A highly potent competitive inhibitor of hydroxymethylglutaryl-coenzyme A reductase and a cholesterol-lowering agent. Proc. Natl. Acad. Sci. USA.

[233] Moore K.S., Wehrli S., Roder H., Rogers M., Forrest Jr J.N., McCrimmon D., Zasloff M. (1993). Squalamine: An aminosterol antibiotic from the shark. Proc. Natl. Acad. Sci. USA.

[234] Selinsky B.S., Smith R., Frangiosi A., Vonbaur B., Pedersen L. (2000). Squalamine is not a proton ionophore. Biochim. Biophys. Acta (BBA)-Biomembr..

[235] Guttuso Jr T., Andrzejewski K.L., Lichter D.G., Andersen J.K. (2019). Targeting kinases in Parkinson’s disease: A mechanism shared by LRRK2, neurotrophins, exenatide, urate, nilotinib and lithium. J. Neurol. Sci..

[236] Braithwaite S.P., Voronkov M., Stock J.B., Mouradian M.M. (2012). Targeting phosphatases as the next generation of disease modifying therapeutics for Parkinson’s disease. Neurochem. Int..

[237] Braithwaite S.P., Stock J.B., Mouradian M.M. (2012). α-Synuclein phosphorylation as a therapeutic target in Parkinson’s disease. Rev. Neurosci..

[238] Bell R., Vendruscolo M. (2021). Modulation of the Interactions Between alpha-Synuclein and Lipid Membranes by Post-translational Modifications. Front. Neurol..

[239] Lee K.-W., Chen W., Junn E., Im J.-Y., Grosso H., Sonsalla P.K., Feng X., Ray N., Fernandez J.R., Chao Y. (2011). Enhanced phosphatase activity attenuates α-synucleinopathy in a mouse model. J. Neurosci..

[240] Cai Z., Xu J., Xue S., Liu Y., Zhang Y., Zhang X., Wang X., Wu F., Li X. (2015). The E3 ubiquitin ligase seven in absentia homolog 1 may be a potential new therapeutic target for Parkinson’s disease. Neural Regen. Res..

[241] Games D., Valera E., Spencer B., Rockenstein E., Mante M., Adame A., Patrick C., Ubhi K., Nuber S., Sacayon P. (2014). Reducing C-terminal-truncated alpha-synuclein by immunotherapy attenuates neurodegeneration and propagation in Parkinson’s disease-like models. J. Neurosci..

[242] Tavassoly O., Yue J., Vocadlo D.J. (2021). Pharmacological inhibition and knockdown of O-GlcNAcase reduces cellular internalization of α-synuclein preformed fibrils. FEBS J..

[243] Lee B.E., Kim H.Y., Kim H.-J., Jeong H., Kim B.-G., Lee H.-E., Lee J., Kim H.B., Lee S.E., Yang Y.R. (2020). O-GlcNAcylation regulates dopamine neuron function, survival and degeneration in Parkinson disease. Brain.

[244] Li P., Song C. (2022). Potential treatment of Parkinson’s disease with omega-3 polyunsaturated fatty acids. Nutr. Neurosci..

[245] Kirsch M., De Groot H. (2001). NAD (P) H, a directly operating antioxidant?. FASEB J..

[246] Higdon J. (2003). An Evidence-Based Approach to Vitamins and Minerals Health Benefits and Intake Recommendations.

[247] Schoeler M., Caesar R. (2019). Dietary lipids, gut microbiota and lipid metabolism. Rev. Endocr. Metab. Disord..

[248] Mao X.-Y., Yin X.-X., Guan Q.-W., Xia Q.-X., Yang N., Zhou H.-H., Liu Z.-Q., Jin W.-L. (2021). Dietary nutrition for neurological disease therapy: Current status and future directions. Pharmacol. Ther..

[249] Zhao B. (2009). Natural antioxidants protect neurons in Alzheimer’s disease and Parkinson’s disease. Neurochem. Res..

